# The development of common data elements for a multi-institute prostate cancer tissue bank: The Cooperative Prostate Cancer Tissue Resource (CPCTR) experience

**DOI:** 10.1186/1471-2407-5-108

**Published:** 2005-08-21

**Authors:** Ashokkumar A Patel, André Kajdacsy-Balla, Jules J Berman, Maarten Bosland, Milton W Datta, Rajiv Dhir, John Gilbertson, Jonathan Melamed, Jan Orenstein, Kuei-Fang Tai, Michael J Becich

**Affiliations:** 1 Department of Pathology, Center for Pathology Informatics, Benedum Oncology Informatics Center, University of Pittsburgh, Pittsburgh, PA, USA; 2 Department of Pathology, University of Illinois-Chicago, Chicago, IL, USA; 3 Cancer Diagnosis Program, National Cancer Institute, Bethesda, MD, USA; 4 Department of Pathology, New York University, New York, NY, USA; 5 Departments of Environmental Medicine and Urology, New York University, New York, NY, USA; 6 Departments of Pathology and Urology, Emory University, Atlanta, GA, USA; 7 Department of Pathology, George Washington University, Washington, DC, USA; 8 Bioinformatics Program, Human and Molecular Genetics Center, Medical College of Wisconsin, Milwaukee, WI, USA

## Abstract

**Background:**

The Cooperative Prostate Cancer Tissue Resource (CPCTR) is a consortium of four geographically dispersed institutions that are funded by the U.S. National Cancer Institute (NCI) to provide clinically annotated prostate cancer tissue samples to researchers. To facilitate this effort, it was critical to arrive at agreed upon common data elements (CDEs) that could be used to collect demographic, pathologic, treatment and clinical outcome data.

**Methods:**

The CPCTR investigators convened a CDE curation subcommittee to develop and implement CDEs for the annotation of collected prostate tissues. The draft CDEs were refined and progressively annotated to make them ISO 11179 compliant. The CDEs were implemented in the CPCTR database and tested using software query tools developed by the investigators.

**Results:**

By collaborative consensus the CPCTR CDE subcommittee developed 145 data elements to annotate the tissue samples collected. These included for each case: 1) demographic data, 2) clinical history, 3) pathology specimen level elements to describe the staging, grading and other characteristics of individual surgical pathology cases, 4) tissue block level annotation critical to managing a virtual inventory of cases and facilitating case selection, and 5) clinical outcome data including treatment, recurrence and vital status. These elements have been used successfully to respond to over 60 requests by end-users for tissue, including paraffin blocks from cases with 5 to 10 years of follow up, tissue microarrays (TMAs), as well as frozen tissue collected prospectively for genomic profiling and genetic studies. The CPCTR CDEs have been fully implemented in two major tissue banks and have been shared with dozens of other tissue banking efforts.

**Conclusion:**

The freely available CDEs developed by the CPCTR are robust, based on "best practices" for tissue resources, and are ISO 11179 compliant. The process for CDE development described in this manuscript provides a framework model for other organ sites and has been used as a model for breast and melanoma tissue banking efforts.

## Background

Since the completion of the human genome project, there has been a paradigm shift in the way biorepositories have been utilized. Recent advances in the fields of genomics and proteomics are providing novel ways of producing experimental data using biospecimens. This shift has lead to the development of robust clinical annotations for the collected tissues, which easily allows comparative research and in-depth analysis of data among multiple institutions. This new paradigm is further exemplified in 2003 by the RAND Corporation's report on Human Tissue Repositories that recommended "...the collection of consistent and high-quality data associated with every biospecimen and employing a standardized set of common data elements...;" for annotation as a best practice [1].

Common data elements (CDEs) are annotations that are collected in a uniform manner across multiple institutions that allow sharing of data in a standardized format and are defined in detail using a metadata dictionary.

In 1999 the National Cancer Institute (NCI), recognizing the need for a multi-center effort in prostate cancer tissue banking, issued an RFA for a consortium effort to collect large numbers of clinically annotated prostate cancer specimens for the research community [2]. This initiative was created after a similar successful NCI Resource that was created for breast tissue called the Cooperative Breast Cancer Tissue Resource (CBCTR) [3,4]. In April 2000, four academic institutions were funded by the NCI to form a national prostate cancer tissue resource, the Cooperative Prostate Cancer Tissue Resource (CPCTR). The goal of the CPCTR is to collect large numbers of prostate cancer specimens with accurate quality controlled and standardized pathologic review of specimens and detailed, quality controlled outcome data for use in biomarker validation studies, and to make this collection available to the research community. During the initial phase of this project, many of the experiences and basic infrastructural components of the CBCTR were used as a model in developing the CPCTR program. Specifically, the CBCTR data elements were used by the CPCTR team to create common data elements (CDEs) for annotating the archival paraffin embedded tissue samples in the prostate resource; the CBCTR does not include frozen tissue collection.

The process of developing CDEs typically involves many individuals and can take up to several months to arrive at a draft that is based on complete consensus among those involved. In the case of CPCTR there were pathologists, urologists, cancer registrars, data managers, and cancer researchers from five major medical centers and the NCI Cancer Diagnosis Program who provided input and approved changes to the developing CDEs along the process of adopting the initial version. In this process it was essential to 1) include experts from multiple disciplines, 2) consider the works of others creating similar CDEs, and/or 3) consider established standards when available. This communication describes the process of developing CDEs for prostate cancer tissues that are banked by the NCI's CPCTR [5,6].

## Methods

### Participating institutions

The Resource comprises four academic institutions: George Washington University Medical Center (GWU), Washington, DC; Medical College of Wisconsin (MCW), Milwaukee, WI; New York University School of Medicine (NYU), New York, NY; the University of Pittsburgh (PITT), PA. The Resource has access to cases from a variety of medical care settings that include academic medical centers, as well as private, public, and Veterans Administration hospitals. The participating hospitals are distributed across six states in the Northeastern and Midwestern regions of the US. This varied access to cases allows accrual of cases that reflect a wide diversity of patients undergoing prostate cancer management in the United States.

### Human subjects protections

The CPCTR uses a decentralized sample and data collection and storage with a centralized data management repository model. Each CPCTR institution has developed its own local protocols with including consent language describing its procedure to protect the confidentiality and privacy of human subjects and has obtained local IRB approval for all CPCTR activities. Tissue data records from the cooperating institutions are submitted to a central data manager (Information Management Services, Inc. (IMS, Bethesda, MD) [16], contracted by the NCI). All institutions assign a random, ten-digit number generated by IMS to each record before submitting the data to the central database. The only linkage to patient identity is retained locally at each CPCTR institution. This ensures that the central database has no links connecting records to patients. In addition, de-identified datasets are generated from the central database for the research community to query (the so-called safe harbor approach to HIPAA-compliance) [7]. The ranges of ages are provided instead of the date of birth and diagnosis to meet the compliant requirements and research purposes.

### Organization of the Resource

**Figure 1:**
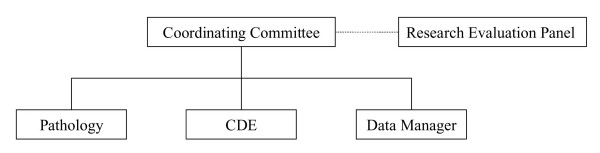
**CPCTR Organization of the Resource**. The Coordinating Committee determines the types of biospecimens the CPCTR will provide. The Research Evaluation Panel (REP) from NCI is the committee in consultation with the Coordinating group. The sub-committees, pathology, CDE, data manager coordinate each other to develop the CDEs for different types of biospecimens that CPCTR will collect.

The CPCTR is governed by a Coordinating Committee that has delegated tasks to several sub-committees. Figure 1 describes the three sub-committees that are involved in the CDE development. The Coordinating Committee includes the four principal investigators (PIs), four co-PIs, a biostatistician, the NCI program leader, two central database coordinators, and a member of the Research Evaluation Panel (REP) that reviews request for tissues and data by end-users. The Committee's main function is to oversee all of the activities of the CPCTR. The Committee's role in developing the CDEs was to determine the types of biospecimens (i.e. paraffin archival tissue, frozen tissue, TMAs) the CPCTR will provide. The details of the CPCTR organization are described in the Resource Manual of Operations located at one of the Resource's websites [8].

The pathology sub-committee includes at least one genitourinary (GU) pathologist per member institution, a NCI program leader, and ad hoc urologists and/or prostate cancer researchers. Their major role is to develop standard evaluation guidelines and propose pathology-specific CDEs related to the different types biospecimens collected to the CDE sub-committee.

The data manager sub-committee includes data managers and cancer registrars from each of the four member institutions as well as the CPCTR biostatistician, two central database coordinators, and the NCI program leader. This sub-committee's main role is to implement and evaluate the CDEs and to perform quality assurance checks on the data collected at each member institution and to help coordinate the distribution of tissue requests and the associated data sets.

The CDE sub-committee includes multiple members from each of the previously mentioned committees. This sub-committee's role was to develop CDEs described in the following section.

### Development of the Common Data Elements

With guidance from the Coordinating Committee and the other sub-committees, the CDE sub-committee's primary tasked was to develop CDEs for demographics and clinical history, specimen level annotation describing the overall case where a bio-specimen was collected, block level annotation which records information on individual pieces or sections of the bio-specimen banked, and follow-up information about treatment, vital status, biochemical (prostate specific antigen [PSA] values) and clinical recurrence to be included in the database. While utilizing and learning from the experiences of several others groups, the CDE sub-committee particularly took the experiences of the CBCTR into considerations [3,4]. The sub-committee also considered established open source standards including the AJCC Cancer Staging Manual [9], the NAACCR Data Standards for Cancer Registries [10], the CAP Cancer Checklist [11], and other prostate specific CDEs that were available through the NCI Center for Bioinformatics (NCICB) [12,13].

### Development of metadata for CDEs

**Figure 2:**
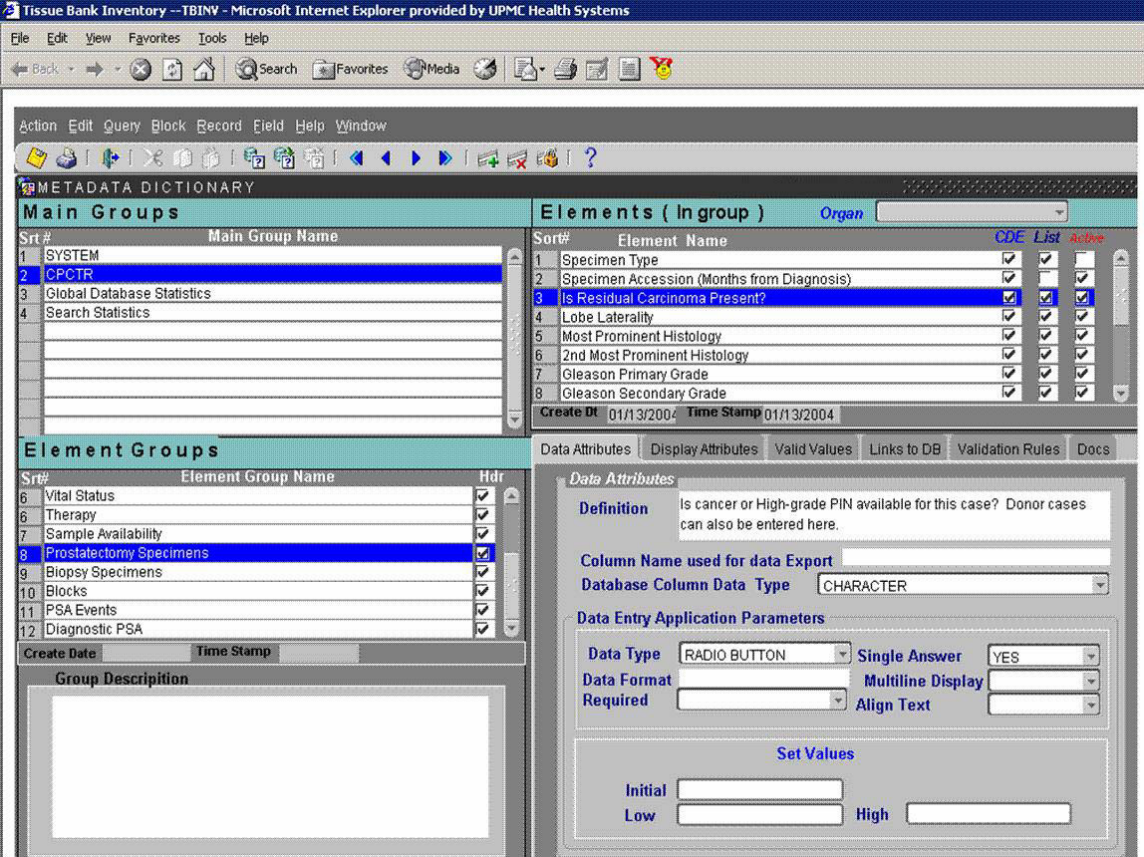
**CPCTR metadata dictionary application**. This is a screen shot of the Oracle mid-tier application used for adding or modifying active CDEs along with the associated metadata. It is also used for generating the CPCTR public query tool available at

Metadata is additional data developed to describes a specific CDE by following the ISO 11179 standard, which "specifies that metadata should have a qualified name or identifier, an authority who registers the name, a versioning history (allowing for modifications), a language or origin, a statement relating to usage, a data typing statement, and a definition that is unambiguous [14]." The CPCTR data dictionary describing each of the common data elements was generated by following the ISO-11179 standard for meta-data. The most current version of the CDE data dictionary can be accessed at the CPCTR public database website [15]. This version of the documentation was generated by the implementation of Oracle's Application Server (v9.0.2) on a Compaq DL360 Server running Windows 2000 with Service Pack 2. The application, as shown on figure 2, uses the Oracle http server and mod_plsql extensions to generate dynamic pages from the database to the users.

### Development of the CPCTR database

Once the initial set of CDEs was developed and approved by the Coordinating Committee, it was used to create a Microsoft Access database by IMS [16]. This database was then distributed to allow each of the member institutes to capture data on all of the tissue samples they will provide to the Resource. Each member institute either utilized this Access database or developed its own database based on it utilizing the technologies that fit its own institutional development environment. Two of the member institutions created their own database using Oracle, while the two other institutions modified the Access database to collect other data elements unique to their local biospecimen collection efforts. By following each institutional IRB's approved protocol, data were collected in the local database. Although, they use different databases, data for all the CDEs from each case were exported to the IMS central database on a monthly basis in pre-defined formats in excel worksheets with the IMS identifier that was randomly generated and pre-assigned to each institution. Once imported into the central database, data QA checks are conducted to detect any missing essential CDEs or possible data input errors including field and cross-field checking (i.e. number of nodes positive >1, then pathology nodal stage = pN1). The valid field options for each data element are defined in the CDE description. Any records with invalid or discrepant data items are censored (i.e., removed from the available tissue samples for investigators) so they are not selected for an application request until they are resolved. Resolutions are the responsibility of the sending institution and are repaired and re-sent with the next monthly data update. In addition, after the initial implementation of any newly created CDEs, there is a short pilot phase where the IMS data check quality assurance review catches any errors and notifies the local institute to resolve the problem.

Furthermore, all of the HIPAA's proscribed set of 18 data elements was omitted from sample records to create a public database [17] for the research community to use. The de-identified data were also utilized by the IMS for filling tissue disbursement to end-users.

### Evaluation of the CDEs

The evaluation phase of the CDEs allowed the CPCTR to examine the quality of data collected by each of the member institutions. The evaluation, an ongoing effort, is carried out at multiple levels by the data manager's sub-committee and reported to the Coordinating Committee. As previously mentioned, the initial evaluation is conducted once any specific CDE is approved and changes to Access database are made. A test export file is sent to IMS to verify correct implementation of any new updates. IMS performs a data check for accuracy and completeness and notifies the local institution of any issues to resolve. The second evaluation, conducted on a monthly basis, is the one carried out by the Data manager's sub-committee (QA checks on 10% of all new cases entered into their local database and sent to IMS).

Finally, the data managers also re-evaluated all CDEs in the entire central database once an initial benchmark of 2,000 cases submitted into the central database was reached. The CDE sub-committee's tasks in this final effort were to determine which CDEs were least populated with valid values (e.g., the CDE "patient's history of other cancers"), which valid values were least used to populate a particular CDE (e.g., the CDE "vital status" has valid values of 'alive, alive with prostate cancer, dead, dead with prostate cancer, dead with autopsy, dead with warm autopsy'), and which CDEs created difficulties in collection (e.g., distant site 1 at the time of diagnosis, date of 1^st ^recurrence, 1^st ^non-prostate recurrence, and distant site of 1^st ^recurrence). Standards for the discontinuation, consolidation, or expansion of CDEs were decided on by committee consensus after discussion and review. Individual institutions could choose to keep discontinued CDEs locally if they were associated with specific institutional research goals.

## Results

### Inventory of resources for CPCTR

At each institution, archival specimens from radical prostatectomies, diagnostic needle biopsies, and surgically removed metastatic tissue specimens from 1989 to present were identified from the pathology records. At the time of compilation of this manuscript (October 2004), there were more than 6,000 annotated cases of prostate cancer specimens with data on over 30,000 archival paraffin-embedded tissues blocks and 5,800 frozen tissue blocks, and two sets of tissue microarrays (TMAs) that are currently available to the research community. The majority of these cases consisted of archival paraffin blocks from surgical patients treated between 1989 and 1998. The remaining cases are recent cases (accrued from 1999 onwards) with prospectively banked tissue (both frozen and paraffin embedded tissue). At some CPCTR sites (e.g. Medical College of Wisconsin, George Washington University, and University of Pittsburgh), blood, serum, and urine samples have also been collected prior to or at the time of surgery from prospectively banked radical prostatectomy patients. The Resource has also accrued diagnostic needle biopsy specimens from at least 2,209 of the radical prostatectomy patients that are entered into the Resource and from 940 prostate cancer patients who did not undergo a radical prostatectomy. The latter samples represent patients who were not eligible for prostatectomy, and received radiation or hormonal therapy, underwent watchful waiting or have died from other causes including other cancers.

### Development of the CDEs

**Figure 3:**
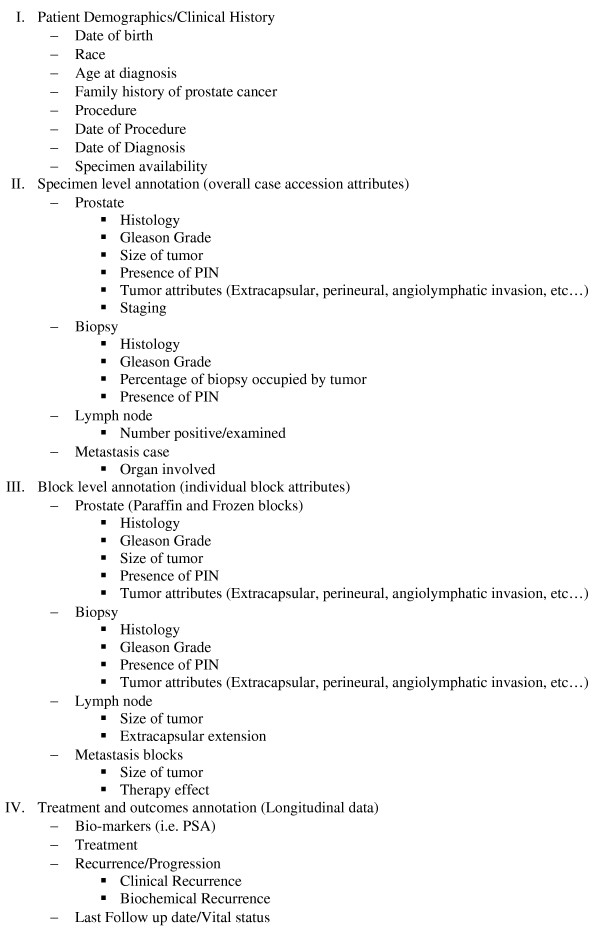
**CPCTR CDE categories**. Four main categories of CDEs and the sub-data types collected. Detail description of each CDE under these four main categories and its sub-groups can be found in the CPCTR CDE data dictionary.

The Coordinating Committee [18] created four main data categories for annotation of the types of specimens banked as a guideline for the CDE development process. The four main categories were: 1) Patient demographics and clinical history data; 2) Specimen annotation, which records basic overall information on a particular event where a bio-specimen was collected as a result of a clinical intervention and/or a specific banking event for research based on a protocol; 3) Block level annotation which records attributes detailing each specimen's paraffin or frozen tissue block entered into the Resource (so-called "matrix blocks") from a particular case; and 4) Treatment and outcomes annotation, which records data that is collected in a longitudinal manner through an "event table" so that outcomes based research can be performed. Sub-categories and additional data elements for each of the four main groups are described in figure 3.

The CDE sub-committee identified and developed CDEs within each of the four main categories by reviewing data elements created by other existing consortiums and open sources. Specifically, the CDE sub-committee used the CBCTR core model and expanded it to include detailed block level annotation, including multiple types of tissue [i.e. prostatectomy (frozen and paraffin), biopsy, lymph node, metastasis]. In addition, the CDE sub-committee developed a set of data elements to address clinical outcome by expanding treatment and recurrence fields to include initiation and completion dates so that a clinical timeline of major events can be followed over time for a patient's course of disease. These annotations importantly include the prostate tumor serum marker (PSA) critical to determining biochemical recurrence. The group also used established standards from the American Joint Commission on Cancer (AJCC) for the staging data elements, the College of American Pathologists (CAP) check list for the annotation at a specimen level, and some elements from the North American Association of Central Cancer Registries (NAACCR) for demographics and follow up data elements [9-11].

### Common Data Elements

The Resource database consists of a set of 145 common data elements (CDEs) which capture the clinical, pathologic and tissue sample inventory data for each case. The data set for the biopsy and metastatic specimens from patients who did not undergo surgery includes many of the radical prostatectomy CDEs with slight variations on individual block descriptors. Each of the data elements is fully described as a set of features conforming to the ISO-11179 standard for meta-data [14]. The data dictionary detailing these CDEs and the associated paper data forms, used by the pathologists and data managers for capturing data, is included as an attachment to this article. The most current version of the approved CDEs can be found on the CPCTR public database website [15].

In order to facilitate material tracking and the identification of specimen-specific characteristics needed during tissue send out or processing, the Resource captures the pathology characteristics of the tissue specimens at the level of individual paraffin tissue blocks and frozen tissue slices for each case. These annotations were named "block matrix" because the annotations are spreadsheet-like in character. From each case the "block matrix" was applied to paraffin blocks of cancer that were selected for inclusion in the Resource collection, with annotation for each block of histologic tumor type, size of tumor focus, Gleason grade, presence of high grade prostatic intraepithelial neoplasia (HGPIN), and presence of perineural, seminal vesicle, or lymphovascular invasion. A detailed annotation is also used for a lymph node matrix block of cases where metastatic tumor was identified in lymph nodes removed at the time of radical prostatectomy. The two other types of matrix blocks annotated by the Resource are blocks containing HGPIN (but no cancer), and benign prostate tissue from areas adjacent to tumor containing prostatectomy blocks, which may be used as one form of "control" tissue.

**Figure 4:**
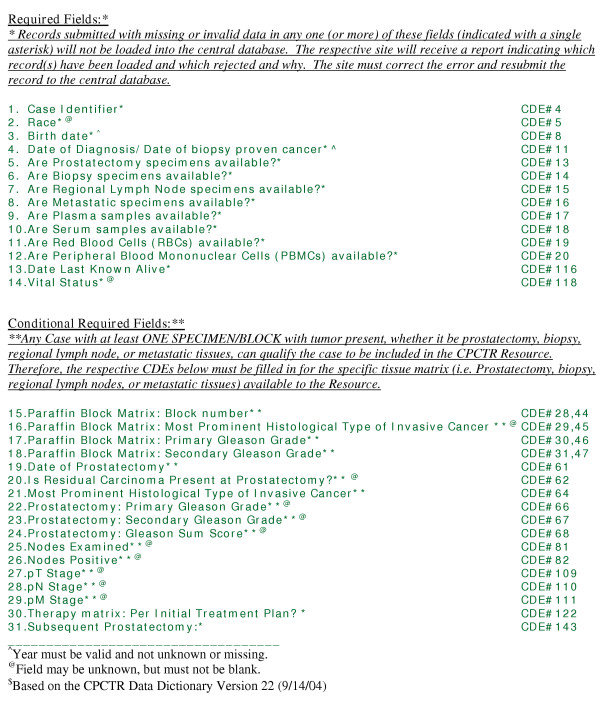
**CPCTR Critical Data Fields**. The critical data fields are divided into two categories^$^: 1) Required fields, which are essential and must be entered into the database for a case to be accepted. 2) Conditional required fields, which must be filled out when the respective tissue matrix is entered into the Resource.

The CDE sub-committee also determined that 14 of the CDEs developed were critical (or 'required') data fields, listed on figure 4. These critical data items are the minimum data elements required for a case to be eligible for inclusion in the Resource. Any records with missing or invalid critical data items are rejected and the corresponding institution is responsible for resolving the issue. In additional, there are 17 "conditional" required data items.

### Re-evaluation of CDEs

**Figure 5:**
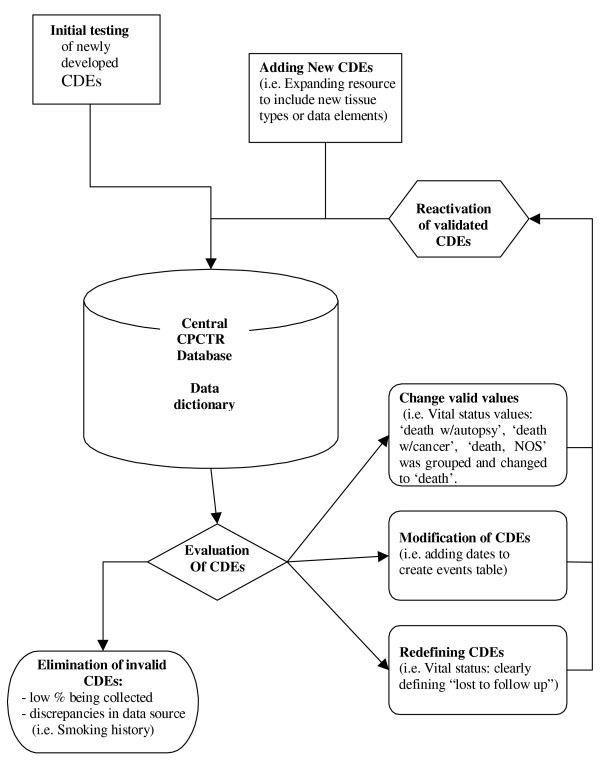
**Re-Evaluation of CDEs**. The flow chart describes the re-evaluation process involved for validating the CDEs. Any changes made after this process, usually eliminates discrepancies and difficulties with the data collection process. In addition, the database also shows an increase in number of fields being populated with valid values and a decrease in "unknown" values.

Annual re-evaluation of the CDEs by the data managers of the CPCTR is conducted to determine which CDEs are most useful for routine tissue and data collection and for long-term updates by all the sites. An overview of the process is described in figure 5. If any desired CDE was found to be poorly collected from a quality control or practical standpoint, discussions were initiated to modify the data collection process through discussions with the cancer registrars and data managers. For example, the evaluation of the data collected for recurrence and progression showed that the initial definitions for CDEs caused difficulties for the data managers collecting this information prompting re-definition of those CDEs. The definitions of the CDEs and their metadata were found to be critical in the clear understanding of what information was to be collected. For example, the initial collection of data related to the CPCTR's CDE for distant metastasis and recurrences were entered in multiple fields (i.e., distant metastasis at the time of diagnosis, distant site of 1^st ^recurrence, 1^st ^non-prostate recurrence and metastatic lymph nodes). Review revealed that these data elements were being poorly collected in quantity (<1% of cases with a valid response) and quality (75% cases with discrepancies when compared to clinical staging or PSA recurrence data). A discussion with the cancer registrars revealed inconsistent application of the definitions for distant metastasis and recurrence. Consequently, the CDEs were modified and re-defined, and subsequent re-evaluation of the collected cases revealed improved data collection.

The first generation CDEs that were determined to result in poorly collected data from all the sites were eliminated and no longer collected after a review of the initial 2600 cases entered into the Resource. For example, a CDE for smoking history attempted to collect multiple values such as "current smoker, never smoked, past smoker, smoker (current or past unknown), and unknown". These values were available for only a limited number of cases (44% of cases with data), which many of them were inconsistently applied because of ambiguous definitions and subjective reporting from the clinical charts. These concerns led the CPCTR Coordinating Committee [18] to eliminate this CDE. However, each institution that elected to resume collecting discontinued CDEs were allowed to locally, but were not reported to the central database.

The Coordinating Committee also re-evaluates the resource as a whole periodically to meet the needs of the prostate research community. The CDE sub-committee is charged to add new CDEs based on the needs of the researchers and addition of new resource materials (e.g., biopsy only specimens, frozen matrix blocks, TMAs, and metastatic tissue blocks). The Coordinating Committee has added dates to many of the CDE categories to be able to examine the timeline of major events that may occur for each patient during his course of disease. This "event table" has resulted in a better picture and assessment of the inter-related characteristics of patient treatments and outcomes.

Once the changes of the CDEs were approved, the local and central databases were modified accordingly. The existed data were programmed to be mapped and stored to the updated databases. The local dataset from each site was queried and sent to the central database following the requested format and validated to ensure the correction of the CDEs updating.

## Discussion

In order to develop any biospecimen resource with high quality specimen annotation, the initial process of building the resource involves significant time and commitment from many experts from various disciplines. Open discussions and input from all potential parties with a stake in the outcome is crucial to any such developmental work. The process of developing the CDEs for the CPCTR has attested that this approach can successfully lead to the implementation of robust prostate tissue CDEs that guide the collection of quality data at over 18 different institutions or hospitals [6,19,20].

Success depends on the ability to collect data using CDEs that have been evaluated by a working group that provides inputs from various experts. The CPCTR CDE sub-committee included organ specific clinicians (pathologists and urologists), informaticians, biostatisticians, data managers, cancer registrars, and research scientists. Clinicians were primarily responsible for providing the foundation of data elements as they reflected the current standard of information used in patient care decisions, while attempting to project at least five years into the future for additional data that may become clinically significant. Likewise, research scientists provided input on data elements that would be crucial in the evaluation of current or proposed prostate cancer research with respect to the detection, diagnosis, prognostication, and treatment of prostate cancer. Thus the result was the creation of datasets that should provide value to the research community when requested from the resource for years to come.

Local data collection methods vary at each institution based on personnel. Some sites have cancer registries responsible for obtaining patient follow up data, while others obtain data from their registry systems or have independent nurses or data managers who extract data from Urology offices by reviewing charts. Thus, it was important to include these nurses, data managers, and cancer registrars who are the main data collectors for the tissue banking resource in CDE development. Their input on the types of data and meta-data available for collection proved to be crucial in aggregating highly quality annotation data for the bio-specimens. Moreover, the definitions of the CDEs and their associated metadata need to be clearly understandable to all those who collect data. For example, in order to collect quality data, the collectors need to understand 1) the fundamental definition of the data element (i.e., date of diagnosis), 2) how that data element will be collected (e.g. 11/2003 vs. Nov. 2003 vs. 11/03, etc), 3) what are the consensus acceptable values or codes are for the data element (e.g., precise date of birth, not calculated from clinical records where the "patient appears to be a well developed 75 year old"), and 4) what the acceptable data format is for inclusion into the central database (e.g., dates as integers not character strings). Through the use of ISO 11179 compliance standards, the goals of collecting annotation data of high quality was achievable, and emphasize the consensus approach used by CPCTR as being critical to successful CDE creation. Although the concept of formalized metadata is fairly straight forward, it has been rarely incorporated by clinical and research groups building databases [14].

Another demonstration of the benefit by CPCTR CDEs is evident from the implementation of the TMA data exchange specification sponsored by the Association of Pathology Informatics (API) [21]. This specification has been used to provide a supplemental XML (Extensible Markup Language) file of the data describing each of the cores in the TMA slides provided to researchers through the CPCTR [22]. The CDEs allow the Resource to directly port data elements and associated metadata directly into a TMA file that complies with the API's TMA specification document and that contains a protected namespace for the CPCTR metadata. These study cases show the examples that well developed CDEs can benefit comparative research and in-depth analysis of data among multiple institutions and studies. The only way in which information from multiple databases can truly be shared and made useful is through the careful use of clearly defined metadata and CDEs [23].

Informaticians and database developers provided the structural link that brought the CDEs together in the database, addressed technical issues, and provided guidance related to implementation of the CDEs at local institutions. The success of the CPCTR CDEs is shown by their implementation at four separate institutions using four different databases, thus demonstrating the ease at which these standards can be copied and distributed across institutions. Regardless of which type of database is used locally, each site is responsible for mapping their data dictionary to the CPCTR CDEs when their data is sent to IMS to be shared. At each individual institution additional data is collected that is pertinent to institution-specific research goals. Yet this incorporation of the common CDEs allows institutions to share data and results across groups while maintaining the autonomy of their research objectives.

Furthermore, having the ability to collect high quality "simple" data elements that have been agreed upon by a working group is crucial for the overall quality of quantitative analysis of inter-institutional data. Collecting simple, yet uniform and comprehensive data annotations in a common database for research across multiple institutions, each with various capabilities of collecting the data (manual review of medical charts, cancer registry systems, and interfaces to legacy systems), vastly increases the statistical power of research efforts and has the potential to identify common trends and issues in cancer care. It is critical that these trends and issues be addressed if we are to find methods to reduce the cancer burden and cancer pain and suffering as is the goal of the NCI [24]. The value of tissue banks and the informatics that support these goals are clearly outlined in the NIH and NCI strategic roadmaps [25].

## Conclusion

Recently, there has been an increasing number of international [26-32] as well as national and state-wide [20,33,34] initiatives that have promoted formation of large research consortia and encourage these groups to share both tissue and data. Currently, many tissue banks such as the CBCTR [3,4], CHTN [35], CFR [36], SPOREs [37], EDRN [13,38] and the PCABC [20] involve multiple institutions. These biorepositories vary in their data collection and tissue collection methodologies. However, the necessity for well annotated tissues that can be re-annotated with experimental data has driven many of these multi-institutional collaborations to develop standards of sharing data with other groups. Currently, the CPCTR CDEs are specifically related to the available prostate tissue resources and clinical data, while experimental data generated from these tissue specimens are not required to be submitted to the resource. However, publications resulting from the use of CPCTR tissues are obliged to credit the Resource, allowing the results to be correlated to or compared with other studies using similar CDE standards. Other initiatives such as the Shared Pathology Informatics Network or SPIN [19], the Early Detection Research Network or EDRN [38] and the Cancer Biomedical Informatics Grid initiative or CaBIG [39] can perform follow-up studies by linking their results to CPCTR derived studies by using the common CPCTR CDEs. This also allows for meta-analysis of data across studies through the CPCTR CDEs, resulting in improved statistical power and further detailed analysis. Thus, expanding the CPCTR dataset by combining tissue with experimental data will have tremendous value in enhancing cancer research [40].

Based on the experience of developing CDEs for the CPCTR, the following sequential strategies can be recommended for other research groups involved in future CDE development efforts.

Initial several months to a year:

• Decide what CDEs the resource will need using a committee driven consensus process that include all major stakeholders

• Utilize as a starting point similar CDE initiatives already developed by others and build upon their standards

• Consult a variety of experts, including those that will be collecting the data particularly tissue bankers, cancer registrars and data managers

Next few months:

• Draft a CDE data dictionary which includes not only the structured data, but also precise data field definitions and a consideration of metadata (data that describes the original data)

• Identify the essential/required data elements and ratify them through a consensus process

• Modify or approve CDEs after discussions with all key parties and build consensus among them and any other external experts

• Create corresponding data entry paper forms/data entry interface to central database

Subsequent few months:

• Implement CDEs

• Test/Pilot phase: sharing of data with central database

Continuously ongoing efforts:

• Re-evaluate CDEs and their data values (every year or after set accrual targets)

• Develop quality assurance, quality control and quality improvement protocols to fully develop the CDEs (minimum of once per year quarterly of semi annually or 10% of new data set, which is the current norm for CPCTR)

## Abbreviations

AJCC – American Joint Committee on Cancer

API – Association for Pathology Informatics

CaBIG – Cancer Biomedical Informatics Grid

CAP – College of American Pathologist

CBCTR – Cooperative Beast Cancer Tissue Resource

CDE – Common data elements

CDP – Cancer Diagnosis Program

CFR – Cancer Family Registries

CHTN – Cooperative Human Tissue Network

CPCTR – Cooperative Prostate Cancer Tissue Resource

EDRN – Early Detection Research Network

HGPIN – High-grade prostatic intraepithelial neoplasia

HIPAA – Health Insurance Portability and Accountability Act

HTTP – HyperText Transfer Protocol

ISO – International Organization for Standards

IRB – Institutional Review Board

LN – Lymph nodes

Mets – Metastasis

NAACCR – North American Association of Central Cancer Registries

NCI – National Cancer Institute

NIH – National Institutes of Health

PCABC – Pennsylvania Cancer Alliance Bioinformatics Consortium

PI – Principle Investigator

PSA – Prostate Specific Antigen

QA – Quality assurance

RAND – Rand Corporation, Inc.

REP – Research Evaluation Panel

RFA – Request for Applications

SEER – Surveillance, Epidemiology, and End Results

SPIN – Shared Pathology Informatics Network

SPOREs – Specialized Programs of Research Excellence

TMA – Tissue Microarray

XML – Extensible Markup Language

## Competing interests

The author(s) declare that they have no competing interests.

## Authors' contributions

AAP was the chair for the data manager's sub-committee and wrote the first draft of the manuscript. MJB, who was the chair for the CDE sub-committee and the Coordinating Committee, was responsible for leading the efforts of developing the CDEs. AKB, JJB, MB, MWD, RD, JG, JM, JO, and KFT assisted in the development of the CDEs and incorporation of other existing standards. All authors reviewed and commented on successive drafts of the manuscript and have provided the first author with approval of the final manuscript.

## Pre-publication history

The pre-publication history for this paper can be accessed here:



## Supplementary Material

Additional file 1 **CPCTR CDE dictionary **CPCTR common data elements' data dictionary with full description and meta-data for each of the CDEs. Click here for file

Additional file 2 **CPCTR CDE paper forms. **CPCTR paper forms used for data collection of CDEs by data managers. These forms are used for collecting data at remote locations (i.e. physician offices) and then entered into the database. Click here for file
