# Supplementary material for: The development of common data elements for a multi-institute prostate cancer tissue bank: The Cooperative Prostate Cancer Tissue Resource (CPCTR) experience
Source: BMC Cancer. 2005 Aug 21;5:108. doi: 10.1186/1471-2407-5-108 (PMC1236914; doi:10.1186/1471-2407-5-108)
Supplement: Additional file 1 — CPCTR CDE dictionary CPCTR common data elements' data dictionary with full description and meta-data for each of the CDEs. [file 1471-2407-5-108-S1.pdf]

C P C T R

CPCTR Common Data Elements for Collection of Prostate Cancer Specimens User Instructions Data Dictionary Version 22 (09/14/04)

Document History

4/10/2000	Initial document by MJB from revised Manual of Operations for CBCTR
4/11/2000	Revision by MJB from CPCTR group meeting
4/13/2000	Revision and added excel data element workbook by PJT v4
5/31/2000	Revision by MJB v5 and update of excel data element workbook v5
6/13/2000	Revision by MJB v6 with incorporation of Pathology Committee recommendations
6/13/2000	Revision by MJB v6 elements into groups (demographics, clinical history & progression info, therapy, pathology and block matrix)
6/29/2000	Revision by MJB v7 changes recommended 6/13/00
7/16/2000	Conference call with Jonathan Melamed 7/16/00
7/19/2000	Revision by MJB from Conference call on 7/16/00
8/8/2000	Revision by MJB in response to changes with CPCTR Prostatectomy Form see CPCTR Prostatectomy form MBv4 0808000.doc.
8/9/2000	Revision by MJB to add prostate needle biopsy elements to data form and CDE list and "normal" block elements.
8/24/2000	Revision and margin notes by MJB related to discussions at Coordinating Committee at GWU for prostatectomy and "qualification" of cases.
8/25/2000	Revision and margin notes by MJB related to discussions at Coordinating Committee at GWU for needle biopsies, TURP and metastatic non-prostate tissues.
10/12/2000	Revision from 8/25 meeting notes and short form of CDE for non-central review by PA, data managers
01/25/2001	Revision from 1/18-19 meeting in NY and from conference call on same date
03/22/2001	FINAL REVISIONS to both Prostatectomy and Needle Biopsy for cases with Prostatectomy.
05/10/2001	Revision by MJB from discussions at the Pittsburgh meeting.
07/05/2001	Pre-final revisions by MJB based on teleconference and e-mails
07/15/2002	Revisions added by MWD after review and discussion with AAP.
08/19/2002	Revisions by AAP after data-managers(7/24/02) and CDE committee(8/14/02) conference calls.
09/30/2002	Added logic testing from PITT, MCW. No additional logics from NYU and GWU.
10/24/2003	Approval by Coordinating committee to add new metastatic elements and therapy matrix and drop elements with low reporting data.
03/05/2004	Reformatted complete CDE data dicitionary and revision of ACCESS database to include metastatic specimens and therapy matrix table. Sent to CDE listserver for approval. Data managers conference call (03/11/2004).
09/14/2004	Marking Critical data elements: 1) required CDEs [marked as *] 2)Conditional required fields [marked as **]

CPCTR Participating Institutes

George Washington University Medical Center
Howard University Medical School
Medical College of Wisconsin
New York University
University of Pittsburgh

For additional information regarding the CPCTR common data elements, database, or any of the tissue resources available, please e-mail us at **ASK-CPCTR-L@LIST.NIH.GOV**.

Criteria for inclusion of cases

A. *Any Case with at least ONE SPECIMEN/BLOCK with tumor present, whether it be prostatectomy, biopsy, regional lymph node, or metastatic tissues, can qualify the case to be included in the CPCTR Resource.*

B. **Pathology Criteria for case inclusion in CPCTR Frozen Tissue Prostatectomy archive**

- The only criteria we recommend currently are:

1) At least 1 neoplastic block or more need to be able to be entered into the matrix or case is excluded.

C. Priorities for entering cases in prostatectomy neoplastic block matrix

- These should assist the teams in picking the highest value blocks for the CPCTR archives.
- Since the matrix can include up to 5 blocks here are the recommendations for selection criteria:

1) The first block should include the largest nodule of tumor (as specified by the CDE)
2) The third through fifth blocks should include seminal vesicle invasion, ECE or angiolymphatic invasion (in that order of preference)
3) The second block should include the largest amount of PIN and preferred is an area of PIN that is independent of cancer (if that exists). If the blocks with PIN all also contain cancer that does not exclude them. Try then to pick a block that has the largest amount of PIN.

D. Needle biopsy (in cases with prostatectomy) CDE archive notes

- For needle biopsy cases, the concern was to give the reviewers some guidance on how many blocks to submit and to clarify what happens when there is only one block with tumor. In these cases it was recommended that the institution cut 3 to 5 blanks for their own diagnostic purposes and then make the residual material available to the CPCTR archive
- We decided that a matrix would be set up and leave the number of blocks submitted to the reviewing pathologist, encouraging them to submit as many blocks as possible on a needle biopsy to a maximum of 5. There is no particular order for entering blocks into the needle biopsy matrix.
- Here are the recommended criteria for needle biopsy (in cases with prostatectomy):

1) Can include one block or more (up to 5)
2) Must at least include one block and classify according to the needle biopsy matrix.

Policy Statements

Controlled Dictionaries:

Following the lead of other resources at NCI we will follow a “controlled dictionary” CDE policy. In short, every data element definition will include acceptable data values unless other wise noted. IMS will only test for and process controlled dictionary values.

* Required Items:

Records submitted with missing or invalid data in any one (or more) of these fields (indicated with an asterisk) will not be loaded into the central database. The respective site will receive a report indicating which record(s) have been loaded and which rejected and why. The site must correct the error and resubmit the record to the central database.

**Conditional Required Fields:

Any Case with at least ONE SPECIMEN/BLOCK with tumor present, whether it be prostatectomy, biopsy, regional lymph node, or metastatic tissues, can qualify the case to be included in the CPCTR Resource. Therefore, the respective CDEs below must be filled in for the specific tissue matrix (i.e. Prostatectomy, biopsy, regional lymph nodes, or metastatic tissues) available to the Resource.

Non-required Items:

Records with any errors or invalid data in non-required fields will be flagged. The respective site will receive an error report listing all case numbers with errors along with a description of the errors. Errors should be reconciled and the corrected records resubmitted within 30 days.

Submission of data files to IMS:

Data files will be composed of exported Excel files from the MS-Access data entry tool developed by IMS. IMS will process the data using the policies, variable constraints and logistical tests documented herein. Accepted records will be loaded into the central database. Unacceptable records will not be loaded into the database but returned to the submission site for review and correction. IMS will document the reasons for rejection when the unacceptable records are returned for correction.

Resubmission of records:

Only records that have been corrected should be resubmitted to the central database.

Not Available:

If data element value is not present (non-required elements only), the assumptions are that the data is not available. No special code need be used to define “not available”. Not available is to be interpreted as unknown, not defined, not counted, etc.

Demographics

1. Hospital Identifier

Definition: **Identified ID (i.e. SS# or Medical Record#, etc.) for each case in the Cooperative Prostate Cancer Tissue Resource.**
Data Type: Character String
Table: tbl_Case_Identifiers.str_Hospital_Identifier
Validation Rules:
Comments: This field is NOT EXPORTED to the CPCTR. ONLY THE HOST SITE WILL HAVE ACCESS TO THE LINKAGE INFORMATION. Its purpose is to:

1) Be the primary key in all of the child tables linking to the parent table within the database.
2) Allow each site to index their cases so that they can link back with the de-identified data for follow up and QA purposes.

Value	Value Description
Free Text	Open text field (250 alphanumeric characters)

2. Last Name

Definition: **Last name of patient.**
Data Type: Character String
Table: tbl_CPCTR.str_Last_Name
Validation Rules:
Comments: This field is NOT EXPORTED to the CPCTR. ONLY THE HOST SITE WILL HAVE ACCESS TO THE LINKAGE INFORMATION. Its purpose is to:

1) allow each site to index their cases so that they can link back with the de-identified data for follow up and QA purposes.

Value	Value Description
Free Text	Open text field (250 alphanumeric characters)

3. First Name

Definition: **First name of patient.**
Data Type: Character String
Table: tbl_CPCTR.str_First_Name
Validation Rules:
Comments: This field is NOT EXPORTED to the CPCTR. ONLY THE HOST SITE WILL HAVE ACCESS TO THE LINKAGE INFORMATION. Its purpose is to:

1) allow each site to index their cases so that they can link back with the de-identified data for follow up and QA purposes.

Value	Value Description
Free Text	Open text field (250 alphanumeric characters)

4. Case Identifier*

Definition: **De-identified ID for each case in the Cooperative Prostate Cancer Tissue Resource.**
Data Type: Character String
Table: tbl_Case_Identifiers.str_Hospital_Identifier
Validation Rules: Site: Confirm that ID is from the assigned ID file. If not, reassign and propagate correct ID.
IMS: Compare ID with IMS internal ID file, confirm that ID was assigned to Site.
Comments:
1) IMS will generate unique ID's and distribute to each site. Each site will request more ID's as needed.
2) THIS IS THE DE-IDENTIFIED ID THAT IS SENT TO THE CENTRAL DATABASE/CPCTR.

Value	Value Description
10-Digit alphanumeric	Provided by IMS.

5. Race*

Definition: **Needs to have a positive statement in the med records. Discordant documents should result in further record review. If majority of records specify a specific race, that race should be used. MD's notes (OR reports, H&P, etc.) take priority over hospital notes.**
Data Type: Character String
Table: tbl_CPCTR.str_2_Race
Validation Rules:
Comments: Required CDE. The expected level of unknown cases <10%. We do not want any institution to disqualify more than 20% of their cases on the basis of "required" elements.

Value	Value Description
African American	Includes the designations of Black, Negro or Afro-American.
Asian	Based on birthplace info (i.e. China, Japan, India, or the Philippines) or race reported only as Asian, Oriental, or Mongolian.
Caucasian	White includes Mexican, Puerto Rican, Cuban, and all other Caucasians.
Native American	Native American
Pacific Islander	Pacific Islander
Other	A combination of two or more races OR race not listed above (Specify in Race comment field).
Unknown	Unknown is not known at accessioning

6. Other Race comments

Definition: **Provide further details on "other" race or general**

comments on race field.
Data Type: Character String
Table: tbl_CPCTR.str_16_Race_Comment
Validation Rules:
Comments: Specify all races when 2 or more races are noted.

Value	Value Description
Free Text	Open text field (250 alphanumeric characters)

7. Hispanic Origin

Definition: **Code whether the patient is of Hispanic decent.**
Data Type: Character String
Table: tbl_CPCTR.str_3_Hispanic
Validation Rules:
Comments: Needs to have a positive statement in the medical records. Do not rely on the spelling of name. Some patients can be identified as African American but still have a Hispanic origin. In this case do we assign Hispanic origin as yes. No documentation of Hispanic origin, conclude patient is not of Hispanic decent (unknown). The ROADS guideline recommends that the registrar may consider the patient of Spanish Origin if only documented by surname only or maiden name and there is no contrary evidence that the person is not Hispanic from the medical record.

Value	Value Description
Yes	Yes
No	No
Unknown	Unknown is not known at accessioning

8. Birth date*

Definition: **Record patient's date of birth in month and year found on the patient's pathology report, H&P, admitting sheet.**
Data Type: Integer: MMYYYY
Table: tbl_CPCTR.int_4_Month_of_Birth; tbl_CPCTR.int_5_Year_of_Birth
Validation Rules:
Comments: Birth year is a required field.

Value	Value Description
1-12	2-digit month-Month (MM)
YYYY	4-digit year-Year (YYYY)

9. Family History of Prostate Cancer

Definition: Code whether the patient has a family member with a history of prostate cancer.
Data Type: Character String

Table: tbl_CPCTR.str_8_Family_History_PCA
Validation Rules:
Comments: Documentation can be found in the H&P. Unknown values should also include cases with inconsistent or discordant information. If no information is found which positively states some family member has cancer should be listed as unknown.

**Value**	**Value Description**
Yes	Yes
No	No
Unknown	Unknown is not known at accessioning OR not accessible from usual resources for this information (eg. medical record, registry, chart review, etc)

10. Family History comments

Definition: **Comments related to Family history.**
Data Type: Character String
Table: tbl_CPCTR.str_26_Family_Comment
Validation Rules:
Comments:

**Value**	**Value Description**
Free Text	Open text field (250 alphanumeric characters)

11. Date of Diagnosis/Date of biopsy proven cancer*

Definition: Is the month and year that the primary cancer was histologically confirmed. Gold standard is biopsy date, secondary choices should include biopsy review date, the third choice (not desirable) is the prostatectomy date.
Data Type: Integer: MMYYYY
Table: tbl_CPCTR.int_10_Month_of_Diagnosis; tbl_CPCTR.int_11_Year_of_Diagnosis
Validation Rules: 1. Date of Diagnosis must prior or equal to prostatectomy date (rounded to month).
2. Date of diagnosis must be after Birth date
Comments:

**Value**	**Value Description**
1-12	2-digit month-Month (MM)
YYYY	4-digit year-Year (YYYY)

12. General Demographic Comment Field

Definition: **General Comments related to Demographics.**
Data Type: Character String
Table: tbl_CPCTR.str_18_General_Comment
Validation Rules:

Comments:

Value	Value Description
Free Text	Open text field (250 alphanumeric characters)

SPECIMEN AVAILABLITY

This section will show the types of specimens available through the Resource.

13.Are Prostatectomy specimens available?*

Definition: **Availability of prostatectomy tissue samples.**
Data Type: Character String
Table: tbl_CPCTR.str_Is_Primary_Carcinoma_Tissue_Available
Validation Rules: If "YES", then prostatectomy (paraffin, frozen, or both) block matrix must be filled in.
Comments: Will need to keep this for bookkeeping, i.e. when we exhaust tissue from blocks sent out.

Value	Value Description
No	No
Yes	Yes
Unknown	Unknown

14.Are Biopsy specimens available?*

Definition: **Availability of prostate biopsy tissue samples.**
Data Type: Character String
Table: tbl_CPCTR.str_Biopsy_Blocks_Available
Validation Rules:
1) If "Biopsy-only" case, then this value is "YES" and (*Biopsy-Subsequent prostatectomy*) must be "NO".
2) If "YES", then biopsy block matrix must be filled in.

Comments:
1) Will need to keep this for bookkeeping, i.e. when we exhaust tissue from blocks sent out.
2) NOTE: "Biopsy-Only" cases are cases due to advance disease or other reasons that did NOT lead to a prostatectomy.

Value	Value Description
No	No
Yes	Yes
Unknown	Unknown

15.Are Regional Lymph Node specimens available?*

Definition: **Availability of regional lymph node tissue samples.**
Data Type: Character String
Table:
Validation Rules: If "YES", then regional lymph node block matrix must be filled in.
Comments:
1) Will need to keep this for bookkeeping, i.e. when we exhaust tissue from blocks sent out.

2) *Regional lymph nodes* are of the true pelvis. They are pelvic nodes below the bifurcation of the common iliac arteries and include: Pelvic (NOS), Hypogastric, Obturator, Iliac (internal, external, NOS), and Sacral (lateral, presacral, promontory, NOS) nodes.
3) *Distant lymph nodes* are outside the confines of the true pelvis and their involvement constitutes distant metastasis. They can be imaged using ultrasound, computed tomography, magnetic resonance imaging, or lymphangiography, and include: aortic (para-aortic, periaortic, lumbar), common iliac, inguinal, superficial inguinal (femoral), supraclavicular, cervical, scalene, and retroperitoneal (NOS) nodes.
4) NOTE: Distant Nodes or Regional Nodes removed after a prostatectomy will be entered into the Metastatic Matrix.

Value	Value Description
No	No
Yes	Yes
Unknown	Unknown

16. Are Metastatic specimens available?*

Definition: **Availability of metastatic tissue samples.**
Data Type: Character String
Table: tbl_CPCTR.str_Are_Metastatic_Tissue_Blocks_Available
Validation Rules: If “YES”, then metastatic tissue block matrix must be filled in.
Comments: Will need to keep this for bookkeeping, i.e. when we exhaust tissue from blocks sent out.

Value	Value Description
No	No
Yes	Yes
Unknown	Unknown

17. Are Plasma samples available?*

Definition: **Availability of plasma samples.**
Data Type: Character String
Table: tbl_CPCTR.str_Plasma_Samples_Available
Validation Rules:
Comments: Will need to keep this for bookkeeping, i.e. when we exhaust tissue from blocks sent out.

Value	Value Description
No	No
Yes	Yes
Unknown	Unknown

18. Are Serum samples available?*

Definition: **Availability of serum samples.**
Data Type: Character String

Table: tbl_CPCTR.str_Serum_Samples_Available
Validation Rules:
Comments: Will need to keep this for bookkeeping, i.e. when we exhaust tissue from blocks sent out.

Value	Value Description
No	No
Yes	Yes
Unknown	Unknown

19. Are Red Blood Cells (RBCs) available?*

Definition: **Availability of red blood cell samples.**
Data Type: Character String
Table: tbl_CPCTR.str_RBC_Samples_Available
Validation Rules:
Comments: Will need to keep this for bookkeeping, i.e. when we exhaust tissue from blocks sent out.

Value	Value Description
No	No
Yes	Yes
Unknown	Unknown

20. Are Peripheral Blood Mononuclear Cells (PBMCs) available?*

Definition: **Availability of peripheral blood mononuclear cells.**
Data Type: Character String
Table: tbl_CPCTR.str_PBMC_Samples_Available
Validation Rules:
Comments: Will need to keep this for bookkeeping, i.e. when we exhaust tissue from blocks sent out.

Value	Value Description
No	No
Yes	Yes
Unknown	Unknown

PROSTATIC SPECIFIC ANTIGEN (PSA)

DIAGNOSTIC PSA:

21. PSA value that prompted Diagnostic Biopsy which showed cancer

Definition: **Reported PSA value that is the closest value prior to the diagnostic biopsy date.**
Data Type: Integer
Table: tbl_CPCTR.dbl_12_PSA_Diagnostic_Biopsy
Validation Rules: should be immediately prior to the diagnostic biopsy and *only one value allowed*
Comments: Given the date of diagnosis with a confirmed biopsy, the

diagnostic (pre-biopsy) PSA will be *calculated* as the closest value prior to the diagnostic biopsy date. Add all other (pre/post diagnostic) PSA values in the PSA matrix (see below).

Value	Value Description
Integer	>0;9999, -1 (unknown)

22. Date of PSA that Prompted Diagnostic Biopsy which showed cancer

Definition: **Date when the diagnostic PSA test was reported.**
Data Type: Integer: MMYYYY
Table: tbl_CPCTR.int_14_PSA_Month_that_Prompted_Diagnostic_Biopsy; tbl_CPCTR.int_15_PSA_Year_that_Prompted_Diagnostic_Biopsy
Validation Rules: Month must be between 1 and 12. Enter 00 for unknown.
Comments:

Value	Value Description
1-12, 00	2-digit month-Month (MM); 00 is standard fictionalized value.
YYYY	4-digit year-Year (YYYY); Must enter a valid year.

23. General comments for Diagnositic PSA:

Definition: **Comments related to the diagnositic PSA value.**
Data Type: Character String
Table:
Validation Rules:
Comments:

Value	Value Description
Free Text	Open text field (250 alphanumeric characters)

PSA MATRIX: (Items #24-27 are multiple entry fields)

The PSA matrix section will allow the collection of multiple PSA values that are prior to and after the diagnostic PSA value. We can write scripts to separate the pre-biopsy (for biopsy-only cases)/pre-prostatectomy PSAs from the post-biopsy/pre-prostatectomy PSAs to determine the biochemical recurrence. We can also calculate the diagnostic PSA since we know the biopsy date and all the PSA dates, and will identify the date of the PSA just before the biopsy. Thus, the central database will take the PSA and Date, sort by date, and calculate the needed results. This also allows one to enter in dates in any order (if much later you get an old chart with more values).

24. PSA below threshold (<0.1)

Definition: **Was the PSA value below threshold level of the test**

method performed.
Data Type: Yes/no
Table: tbl_PSA.yes_PSA
Validation Rules:
Comments: In certain older laboratory PSA tests, the threshold of the test might be <0.3.

Value	Value Description
Check box	If box is checked, then the PSA value is below the threshold level of the test, otherwise, value is null.

25.PSA value (pre/post diagnostic only)

Definition: **Collect all PSA values prior to and after the diagnostic PSA value.**
Data Type: Integer
Table: tbl_PSA.dbl_PSA
Validation Rules: Enter a positive number for PSA >=0.1. Enter -1 if unknown.
Comments:

Value	Value Description
Integer	>0;9999, -1 (unknown)

26.Date of PSA

Definition: **Date when PSA test was performed.**
Data Type: Integer: MMYYYY
Table: tbl_PSA.int_Month; tbl_PSA.int_Year
Validation Rules: Month must be between 1 and 12. Enter 00 for unknown.
Comments:

Value	Value Description
1-12, 00	2-digit month-Month (MM); 00 is standard fictionalized value.
YYYY	4-digit year-Year (YYYY); Must enter a valid year.

27.General comments for PSA matrix

Definition: **Comments related to each matrix PSA values.**
Data Type: Character String
Table:
Validation Rules:
Comments:

Value	Value Description
Free Text	Open text field (250 alphanumeric characters)

ANATOMICAL PATHOLOGY GRADING AND CHARACTERISTICS

PARAFFIN BLOCK MATRIX:(Items #28-40 are multiple entry fields. <u>Up to 5 blocks preferred</u>)

The Paraffin block matrix section will allow the collection of block details on cases that meet the minimum requirements for inclusion into the Resource.

NOTE: Priorities for entering cases in prostatectomy neoplastic block matrix

- **These should assist the teams in picking the highest value blocks for the CPCTR archives.**
- **Since the matrix can include <u>up to 5 blocks</u> here are the recommendations for selection criteria:**
  4) **The <u>*first block*</u> should include the largest nodule of tumor (as specified by the CDE)**
  5) **The <u>*third through fifth blocks*</u> should include seminal vesicle invasion, ECE or angiolymphatic invasion (in that order of preference) NOTE: If SV, ECE, AL do not occur, select the next largest area of tumor for 3 to 5 of the matrix.**
  6) **The <u>*second block*</u> should include the largest amount of PIN and preferred is an area of PIN that is independent of cancer (if that exists). If the blocks with PIN all also contain cancer that does not exclude them. Try then to pick a block that has the largest amount of PIN.**

28.Paraffin Block Matrix: Block number**

Definition: **Block number of the block with largest tumor nodule.**
Data Type: Character String
Table: tbl_Block_Matrix.str_MB_Block_Number
Validation Rules:
Comments:

**Value**	**Value Description**
Free text	List surgical pathology block # from case (varies by institution)

29.Paraffin Block Matrix: Most Prominent Histological Type of Invasive Cancer **

Definition: **Most prominent histological type present on block.**
Data Type: Character String
Table: tbl_Block_Matrix.str_MB_Most_Prominent_Histologic_Type_Invasive_Cancer
Validation Rules: If PIN only, then (*Gleason Grading*) should be blank and (*Presence of PIN*) should be checked YES.
Comments:

**Value**	**Value Description**
Adenocarcinoma NOS (aka acinar)	Adenocarcinoma NOS (aka acinar)
Basal cell carcinoma	Basal cell carcinoma

Ductal adenocarcinoma	Ductal adenocarcinoma
Lymphoma	Lymphoma
Mesenchymal tumor (NOS)	Mesenchymal tumor (NOS)
Mucinous adenocarcinoma	Mucinous adenocarcinoma
Neuroendocrine carcinoma	Neuroendocrine carcinoma
PIN only	PIN only
Sarcomatoid carcinoma	Sarcomatoid carcinoma
Signet ring adenocarcinoma	Signet ring adenocarcinoma
Small cell anaplastic carcinoma	Small cell anaplastic carcinoma
Squamous or adenosquamous carcinoma	Squamous or adenosquamous carcinoma
Transitional (not primary)	Transitional (not primary)
Undifferentiated non-small cell carcinoma	Undifferentiated non-small cell carcinoma
Other (specify in comments)	Other (specify in comments)
Unknown	Unknown

30.Paraffin Block Matrix: Primary Gleason Grade**

Definition: **Primary Gleason grade of block**
Data Type: Character String
Table: tbl_Block_Matrix.str_MB_Primary_Gleason_Grade
Validation Rules: If "not adenocarcinoma" then (*Most prominent histological type*) must be either basal cell carcinoma, transitional (not primary), undifferentiated non-small cell carcinoma, sarcomatoid carcinoma, neuroendocrine carcinoma, small cell anaplastic carcinoma, squamous or adenosquamous carcinoma, mesenchymal tumor (NOS), lymphoma, other (specify in comments), or unknown.
Comments:

**Value**	**Value Description**
1	1
2	2
3	3
4	4
5	5
Not adenocarcinoma	Not adenocarcinoma
Not primary tumor	Not primary tumor
PIN only	PIN only
Unknown	Unknown

31.Paraffin Block Matrix: Secondary Gleason Grade**

Definition: **Secondary Gleason grade of block**
Data Type: Character String
Table: tbl_Block_Matrix.str_MB_Secondary_Gleason_Grade
Validation Rules: If "not adenocarcinoma" then (*Most prominent histological type*) must be either basal cell carcinoma, transitional (not primary), undifferentiated non-small cell carcinoma, sarcomatoid

carcinoma, neuroendocrine carcinoma, small cell anaplastic carcinoma, squamous or adenosquamous carcinoma, mesenchymal tumor (NOS), lymphoma, other (specify in comments), or unknown.

Comments:

Value	Value Description
1	1
2	2
3	3
4	4
5	5
Not adenocarcinoma	Not adenocarcinoma
Not primary tumor	Not primary tumor
PIN only	PIN only
Unknown	Unknown

32. Paraffin Block Matrix: Size of Largest Individual Nodule of Invasive Cancer (in cm)

Definition: **Size of invasive cancer present in block.**

Data Type: Double (number)

Table: tbl_Block_Matrix.dbl_MB_Size_of_Largest_Individual_ Nodule

Validation Rules: Enter a positive number. Enter -1 if unknown.

Comments:

Value	Value Description
Number	-1 Or (>=0 And <100) Or is Null

33. Paraffin Block Matrix: Presence of High Grade Prostatic Intraepithelial Neoplasia (PIN)

Definition: **Presence of High Grade PIN in block.**

Data Type: Yes/No

Table: tbl_Block_Matrix.str_MB_PIN

Validation Rules:

Comments:

Value	Value Description
Check box	If box is checked, then the PIN value is Yes, otherwise, value is No.

34. Paraffin Block Matrix: Presence of Extracapsular Extension (ECE)

Definition: **Presence of Extracapsular/Extraprostatic Extension in block.**

Data Type: Yes/No

Table: tbl_Block_Matrix.str_MB_ECE

Validation Rules:

Comments:

Value	Value Description
Check box	If box is checked, then the ECE value is Yes, otherwise, value is No.

35. Paraffin Block Matrix: Presence of Positive Surgical Margin (SM)

Definition: **Positive surgical margin in block.**
Data Type: Yes/No
Table: tbl_Block_Matrix.str_MB_SM
Validation Rules:
Comments:

Value	Value Description
Check box	If box is checked, then the SM value is Yes, otherwise, value is No.

36. Paraffin Block Matrix: Presence of Perineural Invasion (PN)

Definition: **Presence of Perineural Invasion in block.**
Data Type: Yes/No
Table: tbl_Block_Matrix.str_MB_PN
Validation Rules:
Comments:

Value	Value Description
Check box	If box is checked, then the PN value is Yes, otherwise, value is No.

37. Paraffin Block Matrix: Presence of Seminal Vesicle Invasion (SV)

Definition: **Presence of Seminal Vesicle Invasion in block.**
Data Type: Yes/No
Table: tbl_Block_Matrix.str_MB_SV
Validation Rules:
Comments:

Value	Value Description
Check box	If box is checked, then the SV value is Yes, otherwise, value is No.

38. Paraffin Block Matrix: Presence of Angiolymphatic Invasion (AL)

Definition: **Presence of Angiolymphatic Invasion in block.**
Data Type: Yes/No
Table: tbl_Block_Matrix.str_MB_AL
Validation Rules:
Comments:

Value	Value Description
Check box	If box is checked, then the AL value is Yes, otherwise, value is No.

39. Paraffin Block Matrix: Type of Block(s) available

Definition: **Type of block available.**

Data Type: Character String
Table: tbl_Block_Matrix.str_MB_ParFro
Validation Rules:
Comments:

Value	Value Description
Paraffin only	If this value is picked, then only paraffin block is available for this matrix.
Frozen only	If this value is picked, then only frozen block is available for this matrix.
Paraffin and frozen	If this value is picked, then both Paraffin and frozen block is available for this matrix.

40.Paraffin Block Matrix: block comments

Definition: **Comments related to paraffin block matrix.**
Data Type: Character String
Table: tbl_Block_Matrix.str_MB_Comment
Validation Rules:
Comments:

Value	Value Description
Free Text	Open text field (250 alphanumeric characters)

NON-NEOPLASTIC PARAFFIN BLOCK'S:

Try to include at least one block of normal prostate if possible (two blocks are preferred). If it is not possible to find a completely normal block then include one with minimal amounts of PIN or carcinoma. Seminal vesical is not considered a good normal block.

41.Paraffin Prostatectomy Non-Neoplastic Block #1:

Definition: **Block number of non-neoplastic block #1**
Data Type: Character String
Table: tbl_CPCTR.str_155_Prostatectomy_Non_Neoplastic_Block_1_Number
Validation Rules:
Comments: Should be free of PIN or Cancer if possible. Posterior and lateral lobe sections are preferred.

Value	Value Description
Free Text	List surgical pathology block # from case (varies by institution)

42.Paraffin Prostatectomy Non-Neoplastic Block #2:

Definition: **Block number of non-neoplastic block #2**
Data Type: Character String
Table: tbl_CPCTR.str_156_Prostatectomy_Non_Neoplastic_Block_2_Number
Validation Rules:
Comments: Should be free of PIN or Cancer if possible. Posterior and lateral lobe

sections are preferred.

Value	Value Description
Free Text	List surgical pathology block # from case (varies by institution)

43. General Comments on Paraffin Blocks:

Definition: **General comments related to all paraffin blocks.**
Data Type: Character String
Table: tbl_CPCTR.str_20_Matrix_Comment
Validation Rules:
Comments:

Value	Value Description
Free Text	Open text field (250 alphanumeric characters)

FROZEN BLOCK MATRIX: (Items #44-56 are multiple entry fields. Up to 5 blocks preferred)

NOTE: This section is for the FROZEN BLOCK matrix.
It is very possible that some (and occasionally all) of the frozen blocks in the matrix will also have paraffin tissue. When this happens indicate this in data element [Type of Block(s) available]. On occasion, there may be some blocks that are only frozen (site dependent) also indicate this in the aforementioned data items.

The Frozen block matrix section will allow the collection of block details on cases that meet the minimum requirements for inclusion into the Resource.
NOTE: Priorities for entering cases in prostatectomy neoplastic block matrix

- **These should assist the teams in picking the highest value blocks for the CPCTR archives.**
- **Since the matrix can include up to 5 blocks here are the recommendations for selection criteria:**
  1) **The *first block* should include the largest nodule of tumor (as specified by the CDE)**
  2) **The *third through fifth blocks* should include seminal vesicle invasion, ECE or angiolymphatic invasion (in that order of preference) NOTE: If SV, ECE, AL do not occur, select the next largest area of tumor for 3 to 5 of the matrix.**
  3) **The *second block* should include the largest amount of PIN and preferred is an area of PIN that is independent of cancer (if that exists). If the blocks with PIN all also contain cancer that does not exclude them. Try then to pick a block that has the largest amount of PIN.**

44. Frozen Block Matrix: Block number

Definition: **Block number of the block with largest tumor nodule.**
Data Type: Character String
Table: tbl_Frozen_Matrix.str_FM_Block_Number
Validation Rules:
Comments:

Value	Value Description
Free text	List surgical pathology block # from case (varies by institution)

45. Frozen Block Matrix: Most Prominent Histological Type of Invasive Cancer

Definition: **Most prominent histological type present on block.**
Data Type: Character String
Table: tbl_Frozen_Matrix.str_FM_Most_Prominent_Histologic_Type_Invasive_Cancer
Validation Rules: If PIN only, then (*Gleason Grading*) should be blank and (*Presence of PIN*) should be checked YES.
Comments:

Value	Value Description
Adenocarcinoma NOS (aka acinar)	Adenocarcinoma NOS (aka acinar)
Basal cell carcinoma	Basal cell carcinoma
Ductal adenocarcinoma	Ductal adenocarcinoma
Lymphoma	Lymphoma
Mesenchymal tumor (NOS)	Mesenchymal tumor (NOS)
Mucinous adenocarcinoma	Mucinous adenocarcinoma
Neuroendocrine carcinoma	Neuroendocrine carcinoma
PIN only	PIN only
Sarcomatoid carcinoma	Sarcomatoid carcinoma
Signet ring adenocarcinoma	Signet ring adenocarcinoma
Small cell anaplastic carcinoma	Small cell anaplastic carcinoma
Squamous or adenosquamous carcinoma	Squamous or adenosquamous carcinoma
Transitional (not primary)	Transitional (not primary)
Undifferentiated non-small cell carcinoma	Undifferentiated non-small cell carcinoma
Other (specify in comments)	Other (specify in comments)
Unknown	Unknown

46. Frozen Block Matrix: Primary Gleason Grade

Definition: **Primary Gleason grade of block**
Data Type: Character String
Table: tbl_Frozen_Matrix.str_FM_Primary_Gleason_Grade
Validation Rules: If "not adenocarcinoma" then (*Most prominent histological type*) must be either basal cell carcinoma, transitional (not primary), undifferentiated non-small cell carcinoma, sarcomatoid carcinoma, neuroendocrine carcinoma, small cell anaplastic carcinoma, squamous or adenosquamous carcinoma, mesenchymal tumor (NOS), lymphoma, other (specify in

comments), or unknown.
Comments:

Value	Value Description
1	1
2	2
3	3
4	4
5	5
Not adenocarcinoma	Not adenocarcinoma
Not primary tumor	Not primary tumor
PIN only	PIN only
Unknown	Unknown

47. Frozen Block Matrix: Secondary Gleason Grade

Definition: **Secondary Gleason grade of block**
Data Type: Character String
Table: tbl_Frozen_Matrix.str_FM_Secondary_Gleason_Grade
Validation Rules: If "not adenocarcinoma" then (*Most prominent histological type*) must be either basal cell carcinoma, transitional (not primary), undifferentiated non-small cell carcinoma, sarcomatoid carcinoma, neuroendocrine carcinoma, small cell anaplastic carcinoma, squamous or adenosquamous carcinoma, mesenchymal tumor (NOS), lymphoma, other (specify in comments), or unknown.
Comments:

Value	Value Description
1	1
2	2
3	3
4	4
5	5
Not adenocarcinoma	Not adenocarcinoma
Not primary tumor	Not primary tumor
PIN only	PIN only
Unknown	Unknown

48. Frozen Block Matrix: Size of Largest Individual Nodule of Invasive Cancer (in cm)

Definition: **Size of invasive cancer present in block.**
Data Type: Double (number)
Table: tbl_Frozen_Matrix.dbl_FM_Size_of_Largest_Individual_ Nodule
Validation Rules: Enter a positive number. Enter -1 if unknown.
Comments:

Value	Value Description

Number	-1 Or (>=0 And <100) Or is Null

49. Frozen Block Matrix: Presence of High Grade Prostatic Intraepithelial Neoplasia (PIN)

Definition: **Presence of High Grade PIN in block.**
Data Type: Yes/No
Table: tbl_Frozen_Matrix.str_FM_PIN
Validation Rules:
Comments:

Value	Value Description
Check box	If box is checked, then the PIN value is Yes, otherwise, value is No.

50. Frozen Block Matrix: Presence of Extracapsular Extension (ECE)

Definition: **Presence of Extracapsular/Extraprostatic Extension in block.**
Data Type: Yes/No
Table: tbl_Frozen_Matrix.str_FM_ECE
Validation Rules:
Comments:

Value	Value Description
Check box	If box is checked, then the ECE value is Yes, otherwise, value is No.

51. Frozen Block Matrix: Presence of Positive Surgical Margin (SM)

Definition: **Positive surgical margin in block.**
Data Type: Yes/No
Table: tbl_Frozen_Matrix.str_FM_SM
Validation Rules:
Comments:

Value	Value Description
Check box	If box is checked, then the SM value is Yes, otherwise, value is No.

52. Frozen Block Matrix: Presence of Perineural Invasion (PN)

Definition: **Presence of Perineural Invasion in block.**
Data Type: Yes/No
Table: tbl_Frozen_Matrix.str_FM_PN
Validation Rules:
Comments:

Value	Value Description
Check box	If box is checked, then the PN value is Yes, otherwise, value is No.

53.Frozen Block Matrix: Presence of Seminal Vesicle Invasion (SV)

Definition: **Presence of Seminal Vesicle Invasion in block.**
Data Type: Yes/No
Table: tbl_Frozen_Matrix.str_FM_SV
Validation Rules:
Comments:

Value	Value Description
Check box	If box is checked, then the SV value is Yes, otherwise, value is No.

54.Frozen Block Matrix: Presence of Angiolymphatic Invasion (AL)

Definition: **Presence of Angiolymphatic Invasion in block.**
Data Type: Yes/No
Table: tbl_Frozen_Matrix.str_FM_AL
Validation Rules:
Comments:

Value	Value Description
Check box	If box is checked, then the AL value is Yes, otherwise, value is No.

55.Frozen Block Matrix: Type of Block(s) available

Definition: **Type of block available.**
Data Type: Character String
Table: tbl_Frozen_Matrix.str_FM_ParFro
Validation Rules:
Comments:

Value	Value Description
Paraffin only	If this value is picked, then only paraffin block is available for this matrix.
Frozen only	If this value is picked, then only frozen block is available for this matrix.
Paraffin and frozen	If this value is picked, then both Paraffin and frozen block is available for this matrix.

56.Frozen Block Matrix: block comments

Definition: **Comments related to frozen block matrix.**

Data Type: Character String
Table: tbl_Frozen_Matrix.str_FM_Comment
Validation Rules:
Comments:

Value	Value Description
Free Text	Open text field (250 alphanumeric characters)

NON-NEOPLASTIC FROZEN BLOCK'S:

Try to include at least one block of normal prostate if possible (two blocks are preferred). If it is not possible to find a completely normal block then include one with minimal amounts of PIN or carcinoma. Seminal vesical is not considered a good normal block.

57. Frozen Prostatectomy Non-Neoplastic Block #1:

Definition: **Block number of non-neoplastic block #1**
Data Type: Character String
Table: tbl_CPCTR.str_Prostatectomy_Frozen_Non_Neoplastic_Block_1_Number
Validation Rules:
Comments: Should be free of PIN or Cancer if possible. Posterior and lateral lobe sections are preferred.

Value	Value Description
Free Text	List surgical pathology block # from case (varies by institution)

58. Frozen Prostatectomy Non-Neoplastic Block #2:

Definition: **Block number of non-neoplastic block #2**
Data Type: Character String
Table: tbl_CPCTR.str_Prostatectomy_Frozen_Non_Neoplastic_Block_2_Number
Validation Rules:
Comments: Should be free of PIN or Cancer if possible. Posterior and lateral lobe sections are preferred.

Value	Value Description
Free Text	List surgical pathology block # from case (varies by institution)

59. Frozen Tissue: Warm Ischemic Time (in minutes)

Definition: **Turn around time of when tissue was removed from the patient and the time when it was banked.**
Data Type: Double (number)
Table: tbl_CPCTR.dbl_Frozen_Tissue_Time
Validation Rules:
Comments:

Value	Value Description
Number	>0;99, -1 (unknown)

60.General Comments on Frozen Blocks:

Definition: **General comments related to all frozen blocks.**
Data Type: Character String
Table: tbl_CPCTR.str_Frozen_Tissue_Comment
Validation Rules:
Comments:

Value	Value Description
Free Text	Open text field (250 alphanumeric characters)

HISTOLOGICAL CHARACTERISTICS OF PROSTATECTOMY

Enter the overall characteristics of the prostatectomy.

61.Date of Prostatectomy**

Definition: **Record patient's date of prostatectomy in month and year found on the patient's pathology report, H&P, admitting sheet. It can also include the date of cystoprostatectomy.**
Data Type: Integer: MMYYYY
Table: tbl_CPCTR.int_160_Month_of_Prostatectomy; tbl_CPCTR.int_161_Year_of_Prostatectomy
Validation Rules: Date of prostatectomy must be after the diagnostic PSA date, the diagnostic biopsy date and birth date.
Value should be after or equal to date of diagnosis.
Comments: Prostatectomy year is a required field.

Value	Value Description
1-12, 00	2-digit month-Month (MM); 00 is standard fictionalized value.
YYYY	4-digit year-Year (YYYY); Must enter a valid year.

62.Is Residual Carcinoma Present at Prostatectomy?**

Definition: **Is cancer or High-grade PIN available for this case? Donor cases can also be entered here.**
Data Type: Character String
Table: tbl_CPCTR.str_162_Residual_Carcinoma
Validation Rules:
Comments: Will need to keep this for bookkeeping, i.e. when we exhaust tissue from blocks sent out.

Value	Value Description
HGPIN only	HGPIN only
No	Answer NO for prostatectomy done because of BPH or other benign condition.
Yes	Yes

Donor	Donor
Unknown	Unknown

63. Lobe Laterality

Definition: **Prostate lobe origin of this primary prostate cancer.**
Data Type: Character String
Table: tbl_CPCTR.str_164_Lobe_Laterality
Validation Rules: If either one Left, one Right, or one unspecified then pT2b cannot be chosen; If Two chosen, then pT2a cannot be chosen.
Comments:

**Value**	**Value Description**
One left	One left
One right	One right
One unspecified	One unspecified
Two	Two

64. Most Prominent Histological Type of Invasive Cancer**

Definition: **Most prominent overall histological type present in the prostatectomy.**
Data Type: Character String
Table: tbl_CPCTR.str_165_Most_Prominent_Histologic_Type
Validation Rules: If PIN only, then (*Gleason Grading*) should be "PIN only" and (*High Grade PIN*) cannot be "no" or "unknown".
Comments: This is a required CDE.

**Value**	**Value Description**
Adenocarcinoma NOS (aka acinar)	Adenocarcinoma NOS (aka acinar)
Basal cell carcinoma	Basal cell carcinoma
Ductal adenocarcinoma	Ductal adenocarcinoma
Lymphoma	Lymphoma
Mesenchymal tumor (NOS)	Mesenchymal tumor (NOS)
Mucinous adenocarcinoma	Mucinous adenocarcinoma
Neuroendocrine carcinoma	Neuroendocrine carcinoma
PIN only	PIN only
Sarcomatoid carcinoma	Sarcomatoid carcinoma
Signet ring adenocarcinoma	Signet ring adenocarcinoma
Small cell anaplastic carcinoma	Small cell anaplastic carcinoma
Squamous or adenosquamous carcinoma	Squamous or adenosquamous carcinoma
Transitional (not primary)	Transitional (not primary)
Undifferentiated non-small cell carcinoma	Undifferentiated non-small cell carcinoma
Other (specify in comments)	Other (specify in comments)
Unknown	Unknown

65. 2nd Most Prominent Histological Type of Invasive Cancer

Definition: **2nd Most prominent overall histological type present in the prostatectomy.**
Data Type: Character String

Table: tbl_CPCTR.str_166_2nd_Most_Prominent_Histologic_Type
Validation Rules: If PIN only, then (*Gleason Grading*) should be "PIN only" and (*High Grade PIN*) cannot be "no" or "unknown".
Comments:

Value	Value Description
Adenocarcinoma NOS (aka acinar)	Adenocarcinoma NOS (aka acinar)
Basal cell carcinoma	Basal cell carcinoma
Ductal adenocarcinoma	Ductal adenocarcinoma
Lymphoma	Lymphoma
Mesenchymal tumor (NOS)	Mesenchymal tumor (NOS)
Mucinous adenocarcinoma	Mucinous adenocarcinoma
Neuroendocrine carcinoma	Neuroendocrine carcinoma
PIN only	PIN only
Sarcomatoid carcinoma	Sarcomatoid carcinoma
Signet ring adenocarcinoma	Signet ring adenocarcinoma
Small cell anaplastic carcinoma	Small cell anaplastic carcinoma
Squamous or adenosquamous carcinoma	Squamous or adenosquamous carcinoma
Transitional (not primary)	Transitional (not primary)
Undifferentiated non-small cell carcinoma	Undifferentiated non-small cell carcinoma
Other (specify in comments)	Other (specify in comments)
Unknown	Unknown

66. Prostatectomy: Primary Gleason Grade**

Definition: **Primary Gleason grade of prostatectomy.**
Data Type: Character String
Table: tbl_CPCTR.str_167_Gleason_Primary_Grade
Validation Rules: If "not adenocarcinoma" then (*Most prominent histological type*) must be either basal cell carcinoma, transitional (not primary), undifferentiated non-small cell carcinoma, sarcomatoid carcinoma, neuroendocrine carcinoma, small cell anaplastic carcinoma, squamous or adenosquamous carcinoma, mesenchymal tumor (NOS), lymphoma, other (specify in comments), or unknown.
Comments:

Value	Value Description
1	1
2	2
3	3
4	4
5	5
Not adenocarcinoma	Not adenocarcinoma
Not primary tumor	Not primary tumor
PIN only	PIN only
Unknown	Unknown

67. Prostatectomy: Secondary Gleason Grade**

Definition: **Secondary Gleason grade of prostatectomy.**

Data Type: Character String
Table: tbl_CPCTR.str_168_Gleason_Secondary_Grade
Validation Rules: If “not adenocarcinoma” then (*Most prominent histological type*) must be either basal cell carcinoma, transitional (not primary), undifferentiated non-small cell carcinoma, sarcomatoid carcinoma, neuroendocrine carcinoma, small cell anaplastic carcinoma, squamous or adenosquamous carcinoma, mesenchymal tumor (NOS), lymphoma, other (specify in comments), or unknown.
Comments:

**Value**	**Value Description**
1	1
2	2
3	3
4	4
5	5
Not adenocarcinoma	Not adenocarcinoma
Not primary tumor	Not primary tumor
PIN only	PIN only
Unknown	Unknown

68.Prostatectomy: Gleason Sum Score**

Definition: **Gleason sum score of prostatectomy.**
Data Type: Character String
Table: tbl_CPCTR.str_169_Gleason_Sum_Score
Validation Rules:

1) If “not adenocarcinoma” then (*Most prominent histological type*) must be either basal cell carcinoma, transitional (not primary), undifferentiated non-small cell carcinoma, sarcomatoid carcinoma, neuroendocrine carcinoma, small cell anaplastic carcinoma, squamous or adenosquamous carcinoma, mesenchymal tumor (NOS), lymphoma, other (specify in comments), or unknown.
2) If Gleason Sum is 6 or 7 must answer "*Prostatectomy-Percentage of Gleason 4/5 grade (in all of 6's and 7's)*".
3) Sum of character values in (*Prostatectomy-Primary Gleason*) and (*Prostatectomy-Secondary Gleason*) must equal sum of value in (*Prostatectomy-Gleason Sum*).

Comments:

**Value**	**Value Description**
2	2
3	3
4	4
5	5
6	6
7	7
8	8
9	9

10	10
Not adenocarcinoma	Not adenocarcinoma
Not primary tumor	Not primary tumor
PIN only	PIN only
Unknown	Unknown

69. Percentage of Gleason 4/5 grade (in all of 6's and 7's)

Definition: **Percentage of Gleason 4/5 Grade present in the prostatectomy with Gleason sum score of 6 and 7. Underscores prognostic significance of tiny component of Gleason 4 and 5 tumor in Gleason 6 and 7 tumors.**

Data Type: Integer (number)

Table: tbl_CPCTR.int_170_Percentage_Gleason

Validation Rules: If (*Prostatectomy-Percentage Gleason 4/5*) is filled in as greater than 10,

1) then (*Prostatectomy-Gleason Sum*) must have a character value of 6 or 7.
2) And character value in either (*Prostatectomy-Primary Gleason*) and (*Prostatectomy-Secondary Gleason*) or both must include a "Gleason score 4" or a "Gleason score 5" (i.e. choices are 4,4; 4,5; 5,5; 5,4)

Comments:

**Value**	**Value Description**
Integer (%)	-1 Or (>=0 And <=100) Or is Null

70. Size of Largest Individual Nodule of Invasive Cancer (in cm)

Definition: **Size of largest individual nodule of invasive cancer present in the prostatectomy.**

Data Type: Double (number)

Table: tbl_CPCTR.dbl_171_Size_of_Largest_individual_Nodule

Validation Rules:

1) Enter a positive number. Enter -1 if unknown.
2) Size may be unknown (-1) only if pathologic T Stage is known (not "TX"), or if pathologic M Stage is "M1".

Comments:

**Value**	**Value Description**
Number	-1 Or (>=0 And <100) Or is Null

71. Percentage of Gland Occupied by Tumor

Definition: **Percentage of Gland Occupied by Tumor in the prostatectomy.**

Data Type: Character String

Table: tbl_CPCTR.str_172_Percentage_Gland_Occupied_by_Tumor

Validation Rules: Value (~Volume) may be unknown (-1) only if pathologic T Stage is known (not "TX"), or if pathologic M Stage is "M1".

Comments:

**Value**	**Value Description**

<5%	<5%
5 to 25%	5 to 25%
>25%	>25%
Unknown	Unknown

72.Multifocal Disease

Definition: **Multifocal tumors MUST be separated by a certain distance (1-2 cm) so that the chance of artifactual sectioning is eliminated. The INDEX tumor is the primary case and the size is determined from that tumor.**
Data Type: Character String
Table: tbl_CPCTR.str_175_Multifocal_Disease
Validation Rules:
Comments:

Value	Value Description
No	No
Yes	Yes
Unknown	Unknown

73.Presence of High Grade Prostatic Intraepithelial Neoplasia

Definition: **Is High Grade Prostatic Intraepithelial Neoplasia present in the prostatectomy?**
Data Type: Character String
Table: tbl_CPCTR.str_176_High_Grade_Prostatic_Intraepithelial_Neoplasia_Present
Validation Rules:
Comments:

Value	Value Description
No	No
Unknown	Unknown
Yes-focality unknown	Yes-focality unknown
Yes-multifocal	Yes-multifocal
Yes-one or two foci away from the tumor	Yes-one or two foci away from the tumor
Yes-one or two foci in region of tumor	Yes-one or two foci in region of tumor

74.Extraprostatic Extension/Extracapsular Invasion

Definition: **Extraprostatic or Extracapsular extension seen in prostatectomy. Focal Extracapsular extension is defined as less than 0.8mm of ECE and established is >0.8mm. Multifocal is greater than 2 different foci of ECE.**
Data Type: Character String
Table: tbl_CPCTR.str_173_Extraprostatic extension/extracapsular invasion
Validation Rules: If ECE is present in block matrix (paraffin or frozen), then this value must = Focal, Multifocal, or Established.
Comments:

Value	Value Description
Established	Established (>0.8mm)
Focal	Focal (less than 0.8mm)
Multifocal	Multifocal (greater than 2 different foci)
None	None
Unknown	Unknown

75. Surgical Margin Involvement

Definition: **Are Surgical Margins involved by Tumor (ink on margin)?**
Data Type: Character String
Table: tbl_CPCTR.str_174_Surgical_Margins
Validation Rules: If SM is present in block matrix (paraffin or frozen), then this value must = Focal or Widespread.
Comments:

Value	Value Description
All surgical margins are free of tumor	All surgical margins are free of tumor
Tumor focal at margin	Tumor focal at margin
Tumor widespread at margin	Tumor widespread at margin
Unknown	Unknown

76. Presence of Perineural Invasion

Definition: **Is Perineural Invasion present in the prostatectomy?**
Data Type: Character String
Table: tbl_CPCTR.str_177_Perineural_Invasion_Present
Validation Rules: If PN is present in block matrix (paraffin or frozen), then this value = Yes.
Comments:

Value	Value Description
No	No
Yes	Yes
Unknown	Unknown

77. Presence of Seminal Vesicle Invasion

Definition: **Presence of seminal vesical involvement in the prostatectomy.**
Data Type: Character String
Table: tbl_CPCTR.str_178_Seminal_Vesicle_Invasion_Present
Validation Rules: If SV is present in block matrix (paraffin or frozen), then this value = Yes.
Comments:

Value	Value Description
No	No
Yes	Yes
Unknown	Unknown

78. Presence of Angiolymphatic Invasion

Definition: **Is Angio/Lymphatic Invasion present in the prostatectomy?**
Data Type: Character String
Table: tbl_CPCTR.str_179_Angio_Lymphatic_Invasion_Present
Validation Rules: If AL is present in block matrix (paraffin or frozen), then this value = Yes.
Comments:

Value	Value Description
No	No
Yes	Yes
Unknown	Unknown

79. General Comments Section for Prostatectomy:

Definition: **General comments related to prostatectomy.**
Data Type: Character String
Table: tbl_CPCTR.str_182_Comment_for_Prostatectomy
Validation Rules:
Comments: To provide general comments on the entire prostatectomy.

Value	Value Description
Free Text	Open text field (250 alphanumeric characters)

REGIONAL LYMPH NODE STATUS AT THE TIME OF PROSTATECTOMY

Include the lymph node status at the time of the prostatectomy.
-If there is regional lymph node exploration prior or equal to the prostatectomy date, but after the initial diagnostic biopsy, then enter values here (in the Lymph Node section).
-Regional lymph nodes removed <u>after</u> the prostatectomy or Distant lymph nodes should be enter in the *METASTASIS MATRIX* section.
NOTE:
<u>Regional lymph nodes</u> **are of the true pelvis. They are pelvic nodes below the bifurcation of the common iliac arteries and include: Pelvic (NOS), Hypogastric, Obturator, Iliac (internal, external, NOS), and Sacral (lateral, presacral, promontory, NOS) nodes.**
<u>Distant lymph nodes</u> **are outside the confines of the true pelvis and their involvement constitutes distant metastasis. They can be imaged using ultrasound, computed tomography, magnetic resonance imaging, or lymphangiography, and include: aortic (para-aortic, periaortic, lumbar), common iliac, inguinal, superficial inguinal (femoral), supraclavicular, cervical, scalene, and retroperitoneal (NOS) nodes.**

80. Date of Regional Lymph Node resection

Definition: **Record patient's date of regional lymph node resection**

month and year found on the patient's pathology report, H&P, admitting sheet.

Data Type: Integer: MMYYYY

Table: tbl_CPCTR.int_202_Month_of_Lymph_Node_Resection; tbl_CPCTR.int_203_Year_of_Lymph_Node_Resection

Validation Rules: Value should be after or equal to (*date of diagnosis*) and Value should be before or equal to (*date of prostatectomy*).

Comments:

**Value**	**Value Description**
1-12, 00	2-digit month-Month (MM); 00 is standard fictionalized value.
YYYY	4-digit year-Year (YYYY); Must enter a valid year.

81.Nodes Examined**

Definition: **Number of lymph nodes examined at the time of the prostatectomy.**

Data Type: Integer (number)

Table: tbl_CPCTR.int_200_Nodes_Examined

Validation Rules:

1) If > 0, then (*Nodes Positive*) must be >= 0 and (*pathologic N Stage*) must be specified.
2) If "-1", then (*Nodes Positive*) must be "-1" and (*pathologic N Stage*) must be "NX".

Comments:

1) NOTE: -1 = "nodes examined, but number unknown"
2) If no nodes are examined or do not know if any were examined, then enter "0".

**Value**	**Value Description**
Integer	-1 Or (>=0 And <=99) Or Is Null

82.Nodes Positive**

Definition: **Number of positive lymph nodes examined at the time of the prostatectomy.**

Data Type: Integer (number)

Table: tbl_CPCTR.int_201_Nodes_Positive

Validation Rules:

1) If "0", then (*Nodes Examined*) must be >0, and (*pathologic N Stage*) must be "N0".
2) If > 0, then (*Nodes Examined*) must be > 0, and (*pathologic N Stage*) must be "N1".
3) If "-1", then (*Nodes Examined*) must be > 0 or = -1 and (*pathologic N Stage*) must be "NX".

Comments:

1) NOTE: -1 = "*some nodes are positive, but number unknown*"
2) If no nodes are positive or do not know if any were positive, then enter "0".

**Value**	**Value Description**
Integer	-1 Or (>=0 And <=99) Or Is Null

83.Lymph Node Non-Neoplastic Block #1:

Definition: **Block number of non-neoplastic block #1**
Data Type: Character String
Table: tbl_CPCTR.str_Lymph_Node_Nonneoplastic_Block
Validation Rules:
Comments:

Value	Value Description
Free Text	List surgical pathology block # from case (varies by institution)

84.General Comments Section for Lymph Nodes:

Definition: **General comments related to prostatectomy.**
Data Type: Character String
Table: tbl_CPCTR.str_23_Lymph_Comment
Validation Rules:
Comments: To provide general comments on the lymph nodes removed at the time of prostatectomy.

Value	Value Description
Free Text	Open text field (250 alphanumeric characters)

REGIONAL LYMPH NODE BLOCK MATRIX: (Items #85-87 are multiple entry fields. Up to 3 blocks preferred)

NOTE: This section is for the LYMPH NODE BLOCK matrix. Only positive lymph nodes found at the time of the prostatectomy should be entered into the matrix. Lymph nodes removed at later dates should be entered in the METASTASIS BLOCK MATRIX.

85.Lymph Node Block Matrix: Neoplastic Block number:

Definition: **Block number of metastatic lymph node block**
Data Type: Character String
Table: tbl_Lymph_Node_Matrix.str_LNMB_Block_Number
Validation Rules:
Comments: Must be at the time of prostatectomy or Lymph nodes removed at later dates should be entered in the METASTASIS BLOCK MATRIX.

Value	Value Description
Free Text	List surgical pathology block # from case (varies by institution)

86.Lymph Node Block Matrix: Size of Largest Individual Nodule of Invasive Cancer (in cm)

Definition: **Size of invasive cancer present in lymph node block.**

Data Type: Double (number)
Table: tbl_Lymph_Node_Matrix.dbl_LNMB_Size_of_Largest_Individual_ Nodule
Validation Rules: Enter a positive number. Enter -1 if unknown.
Comments:

Value	Value Description
Number	-1 Or (>=0 And <100) Or is Null

87.Lymph Node Block Matrix: Presence of Extracapsular Extension (ECE)

Definition: **Presence of Extracapsular Extension in block.**
Data Type: Yes/No
Table: tbl_Lymph_Node_Matrix.str_LNMB_ECE
Validation Rules:
Comments: Capsule of Lymph Node

Value	Value Description
Check box	If box is checked, then the ECE value is Yes, otherwise, value is No.

RECURRENCE/METASTATSIS STATUS:

The Recurrence/Metastasis Status data elements are separated into three categories:

1) **<u>Biochemical recurrence</u> calls using the PSA recurrence algorithm developed by the CPCTR.**
2) **<u>Clinically verified Tissue recurrence/metastasis</u> for cases, which do not have blocks available for the Resource.**
3) **<u>Metastatic Tissue block matrix</u> for cases that have tissue available through the Resource from anatomical sites that show recurrence/metastasis of prostate cancer.**

BIOCHEMICAL RECURRENCE: (calculated values)

The biochemical recurrence calls are made using the PSA recurrence algorithm developed by the CPCTR. Further details on the algorithm used can be received by contacting the Resource.

88.Biochemical Recurrence Status

Definition: **Biochemical recurrence calls based on the CPCTR PSA recurrence algorithm.**
Data Type: Character String
Table:
Validation Rules:
Comments: This is calculated using the CPCTR PSA algorithm at the Central Database by IMS.

Value	Value Description
Never Disease Free	Residual tumor present
No recurrence	No recurrence
PSA recurrence	Recurred based on CPCTR PSA recurrence algorithm
Unknown	Call cannot be determined using the algorithm

89.Recurrence PSA value

Definition: **PSA value which triggered a recurrence call using the CPCTR PSA recurrence algorithm.**
Data Type: Integer
Table:
Validation Rules: Enter a positive number for PSA >=0.1. Enter -1 if unknown.
Comments: This is calculated using the CPCTR PSA algorithm at the Central Database by IMS.

Value	Value Description
Integer	>0;9999, -1 (unknown)

90.Date of Biochemical Recurrence Status

Definition: **Date when the Recurrence PSA value was reported.**
Data Type: Integer: MMYYYY
Table:
Validation Rules: Month must be between 1 and 12. Enter 00 for unknown.
Comments: This is calculated using the CPCTR PSA algorithm at the Central Database by IMS. If there is no recurrence, then the date last verified recurrence-free date is the same as the last post-prostatectomy PSA value.

Value	Value Description
1-12, 00	2-digit month-Month (MM); 00 is standard fictionalized value.
YYYY	4-digit year-Year (YYYY); Must enter a valid year.

CLINICALLY VERIFIED TISSUE RECURRENCE/METASTASIS:

These cases are those that do not have tissue blocks available, but are known clinically to have a recurrence/metastasis.
NOTE: Verification for clinical recurrence can be via radiology imaging, biopsy/resection, or surgery. However, a clinician's note indicating recurrence in a specific distant site would be sufficient. However this <u>does not include notes of recurrence because of PSA (Biochemical)</u>, note must say "bone, liver, etc. mets".

91.Tissue Recurrence/Metastasis: Distant site 1

Definition: **Location of clinically verified tissue recurrence (site 1).**
Data Type: Character String
Table: tbl_CPCTR.str_221_Distant_Mets_1
Validation Rules:
Comments:

Value	Value Description
Bladder	Bladder
Blood	Blood
Bone	Bone
Bone Marrow	Bone Marrow
Brain	Brain
Carcinomatosis	Carcinomatosis
CNS	CNS
Generalized	Generalized
Liver	Liver
Local extension	Local extension
Lung	Lung
Lymph Nodes (Distant)	Lymph Nodes (Distant)
Lymph Nodes (Regional)	Lymph Nodes (Regional)
Peritoneum	Peritoneum
Rectum	Rectum
Skin	Skin
None	None
Other (NOS)	Other (NOS)
Unknown	Unknown

92. Date of Tissue Recurrence / Metastasis: Distant site 1

Definition: **Date when tissue recurrence is clinically verified and documented in the medical chart.**
Data Type: Integer: MMYYYY
Table: tbl_CPCTR.int_Month_Distant_Mets_1; tbl_CPCTR.int_Year_Distant_Mets_1
Validation Rules:
Comments:

Value	Value Description
1-12, 00	2-digit month-Month (MM); 00 is standard fictionalized value.
YYYY	4-digit year-Year (YYYY); Must enter a valid year.

93. Tissue Recurrence / Metastasis: Distant site 2

Definition: **Location of clinically verified tissue recurrence (site 2).**
Data Type: Character String
Table: tbl_CPCTR.str_223_Distant_Mets_2
Validation Rules:
Comments:

Value	Value Description
Bladder	Bladder
Blood	Blood
Bone	Bone
Bone Marrow	Bone Marrow
Brain	Brain
Carcinomatosis	Carcinomatosis
CNS	CNS
Generalized	Generalized
Liver	Liver
Local extension	Local extension
Lung	Lung
Lymph Nodes (Distant)	Lymph Nodes (Distant)
Lymph Nodes (Regional)	Lymph Nodes (Regional)
Peritoneum	Peritoneum
Rectum	Rectum
Skin	Skin
None	None
Other (NOS)	Other (NOS)
Unknown	Unknown

94. Date of Tissue Recurrence / Metastasis: Distant site 2

Definition: **Date when tissue recurrence is clinically verified and documented in the medical chart.**
Data Type: Integer: MMYYYY
Table: tbl_CPCTR.int_Month_Distant_Mets_2; tbl_CPCTR.int_Year_Distant_Mets_2
Validation Rules:
Comments:

Value	Value Description
1-12, 00	2-digit month-Month (MM); 00 is standard fictionalized value.
YYYY	4-digit year-Year (YYYY); Must enter a valid year.

95. Tissue Recurrence / Metastasis: Distant site 3

Definition: **Location of clinically verified tissue recurrence (site 3).**
Data Type: Character String
Table: tbl_CPCTR.str_225_Distant_Mets_3
Validation Rules:
Comments:

Value	Value Description
Bladder	Bladder
Blood	Blood
Bone	Bone

Bone Marrow	Bone Marrow
Brain	Brain
Carcinomatosis	Carcinomatosis
CNS	CNS
Generalized	Generalized
Liver	Liver
Local extension	Local extension
Lung	Lung
Lymph Nodes (Distant)	Lymph Nodes (Distant)
Lymph Nodes (Regional)	Lymph Nodes (Regional)
Peritoneum	Peritoneum
Rectum	Rectum
Skin	Skin
None	None
Other (NOS)	Other (NOS)
Unknown	Unknown

96. Date of Tissue Recurrence / Metastasis: Distant site 3

Definition: **Date when tissue recurrence is clinically verified and documented in the medical chart.**
Data Type: Integer: MMYYYY
Table: tbl_CPCTR.int_Month_Distant_Mets_3; tbl_CPCTR.int_Year_Distant_Mets_3
Validation Rules:
Comments:

Value	Value Description
1-12, 00	2-digit month-Month (MM); 00 is standard fictionalized value.
YYYY	4-digit year-Year (YYYY); Must enter a valid year.

97. General Comments for Clinically Verified Tissue Recurrence / Metastasis:

Definition: **General comments related to clinically verified recurrence / metastasis.**
Data Type: Character String
Table: tbl_CPCTR.str_27_Recurrence_Comment
Validation Rules:
Comments: To provide general comments on the clinically verified tissue recurrence or metastatic status of patient.

Value	Value Description
Free Text	Open text field (250 alphanumeric characters)

METASTATIC TISSUE BLOCK MATRIX: (Items #98-108 are multiple entry fields):

-Enter as many blocks available (Three blocks are preferred).
-If multiple metastatic sites are present, then enter at least 1 block from each site.
-Try to include at least one block of normal tissue from the same site if possible. If it is not possible to find a completely normal block then include one with minimal amounts of carcinoma.

98.Metastatic Block Matrix: Block number

Definition: **Block number of the block with largest tumor nodule in the metastatic tissue.**
Data Type: Character String
Table: tbl_METS_Matrix.str_METS_Block_ID
Validation Rules:
Comments:

Value	Value Description
Free text	List surgical pathology block # from case (varies by institution)

99.Metastatic Block Matrix: Specimen source

Definition: **Procedure/Method used to acquire specimen.**
Data Type: Character String
Table: tbl_METS_Matrix.str_METS_Specimen_Source
Validation Rules:
Comments:

Value	Value Description
Biopsy	Biopsy
Resection	Resection
Fine Needle Aspirate	Fine Needle Aspirate
Other	Other
Unknown	Unknown

100. Metastatic Block Matrix: Tissue Type

Definition: **Location of clinically verified tissue recurrence (site 3).**
Data Type: Character String
Table: tbl_METS_Matrix.str_METS_Tissue_Type
Validation Rules:
Comments:

Value	Value Description
Bladder	Bladder
Blood	Blood
Bone	Bone

Bone Marrow	Bone Marrow
Brain	Brain
Carcinomatosis	Carcinomatosis
CNS	CNS
Generalized	Generalized
Liver	Liver
Local extension	Local extension
Lung	Lung
Lymph Nodes (Distant)	Lymph Nodes (Distant)
Lymph Nodes (Regional)	Lymph Nodes (Regional)
Peritoneum	Peritoneum
Rectum	Rectum
Skin	Skin
None	None
Other (NOS)	Other (NOS)
Unknown	Unknown

101. Metastatic Block Matrix: Most Prominent Histological Type of Invasive Cancer

Definition: **Most prominent histological type present on metastatic block.**
Data Type: Character String
Table: tbl_METS_Matrix.str_MET_Most_Prominent_Histologic_Type
Validation Rules:
Comments:

Value	Value Description
Adenocarcinoma NOS (aka acinar)	Adenocarcinoma NOS (aka acinar)
Basal cell carcinoma	Basal cell carcinoma
Ductal adenocarcinoma	Ductal adenocarcinoma
Lymphoma	Lymphoma
Mesenchymal tumor (NOS)	Mesenchymal tumor (NOS)
Mucinous adenocarcinoma	Mucinous adenocarcinoma
Neuroendocrine carcinoma	Neuroendocrine carcinoma
PIN only	PIN only
Sarcomatoid carcinoma	Sarcomatoid carcinoma
Signet ring adenocarcinoma	Signet ring adenocarcinoma
Small cell anaplastic carcinoma	Small cell anaplastic carcinoma
Squamous or adenosquamous carcinoma	Squamous or adenosquamous carcinoma
Transitional (not primary)	Transitional (not primary)
Undifferentiated non-small cell carcinoma	Undifferentiated non-small cell carcinoma
Other (specify in comments)	Other (specify in comments)
Unknown	Unknown

102. Metastatic Block Matrix: Size of Largest Individual Nodule of Invasive Cancer (in cm)

Definition: **Size of invasive cancer present in metastatic block.**

Data Type: Double (number)
Table: tbl_METS_Matrix.dbl_METS_Size_of_Largest_Nodule
Validation Rules: Enter a positive number. Enter -1 if unknown.
Comments:

Value	Value Description
Number	-1 Or (>=0 And <100) Or is Null

103. Metastatic Block Matrix: Percentage of Tissue Occupied by Tumor

Definition: **Percentage of Tissue Occupied by Tumor in the metastatic block.**
Data Type: Character String
Table: tbl_METS_Matrix.str_METS_Percentage_Tissue_Occupied_by_Tumor
Validation Rules:
Comments:

Value	Value Description
<5%	<5%
5 to 25%	5 to 25%
>25%	>25%
Unknown	Unknown

104. Metastatic Block Matrix: Presence of Therapy effects

Definition: **Therapy effect: hormonal/radiation effect on tumor**
Data Type: Character String
Table: tbl_METS_Matrix.str_METS_Therapy_Effect
Validation Rules:
Comments:

Value	Value Description
No	No
Yes	Yes
Unknown	Unknown

105. Metastatic Block Matrix: Non-Neoplastic Block number:

Definition: **Block with non-neoplastic tissue associated with the metastatic block submitted above.**
Data Type: Character String
Table: tbl_METS_Matrix.str_METS_Available_NonNeoplastic_Block_ID
Validation Rules:
Comments: List only block number. If not available without tumor then block with largest amount of benign tissue.

Value	Value Description
Free Text	List surgical pathology block # from case (varies by institution)

106. Metastatic Block Matrix: Type of Block(s) available

Definition: **Type of metastatic block available.**
Data Type: Character String
Table: tbl_METS_Matrix.str_METS_Paraffin_AND_Frozen
Validation Rules:
Comments:

Value	Value Description
Paraffin only	If this value is picked, then only paraffin block is available for this matrix.
Frozen only	If this value is picked, then only frozen block is available for this matrix.
Paraffin and frozen	If this value is picked, then both Paraffin and frozen block is available for this matrix.

107. Date of Metastatic Block Matrix:

Definition: **Date when metastatic tissue was removed/excised.**
Data Type: Integer: MMYYYY
Table: tbl_METS_Matrix.str_METS_Month;
tbl_METS_Matrix.str_METS_Year
Validation Rules:
Comments:

Value	Value Description
1-12, 00	2-digit month-Month (MM); 00 is standard fictionalized value.
YYYY	4-digit year-Year (YYYY); Must enter a valid year.

108. General Comments Section for Metastatic Block Matrix:

Definition: **General comments related to metastatic specimens.**
Data Type: Character String
Table: tbl_METS_Matrix.str_METS_Comment
Validation Rules:
Comments: To provide general comments on the metastatic specimens.

Value	Value Description
Free Text	Open text field (250 alphanumeric characters)

STAGING

Pathological and Clinical Staging will be based on using the <u>AJCC Manual for Staging of Cancer, 5th Ed</u>.

PATHOLOGICAL STAGING:

109. pT Stage**

Definition: **Use Manual for Staging of Cancer, 5thEd. Report pT stage at initial diagnosis.**
Data Type: Character String
Table: tbl_CPCTR.str_230_pT_Stage
Validation Rules:
Comments:
1) T0 cases are eligible only for node positive cases where the nodal specimen or metastatic specimen is available and accessioned into the CPCTR.
2) If both clinical and pathologic staging are available, then use pathology.
3) For comments on values below:

*Tumor found in one or both lobes by needle biopsy, but not palpable or reliably visible by imaging, is classified as T1c.
**Invasion into the prostatic apex or into (but not beyond) the prostatic capsule is not classified as T3, but as T2.

Value	Value Description
pT0	No evidence of primary tumor
pT1	Clinically inapparent tumor not palpable nor visible by imaging
pT1a	Tumor incidental histologic finding in 5% or less of tissue resected
pT1b	Tumor incidental histologic finding in more than 5% of tissue resected
pT1c	Tumor identified by needle biopsy (e.g., because of elevated PSA)
pT2	Tumor confined within prostate*
pT2a	Tumor involves 1 lobe
pT2b	Tumor involves both lobes
pT3	Tumor extends through the prostatic capsule**
pT3a	Extracapsular extension (unilateral or bilateral)
pT3b	Tumor invades seminal vesicle(s)
pT4	Tumor is fixed or invades adjacent structures other than seminal vesicles
pTX	Primary tumor cannot be assessed

110. pN Stage**

Definition: **Use Manual for Staging of Cancer, 5thEd. Report pN stage at initial diagnosis.**
Data Type: Character String
Table: tbl_CPCTR.str_231_pN_Stage

Validation
Rules:
Comments:

Value	Value Description
pN0	No regional lymph node metastasis
pN1	Metastasis in regional lymph node or nodes
pNX	Regional lymph nodes cannot be assessed

111. pM Stage**

Definition: **Use Manual for Staging of Cancer, 5thEd. Report pM stage at initial diagnosis.**
Data Type: Character String
Table: tbl_CPCTR.str_232_pM_Stage
Validation
Rules:
Comments: For comments on values:
*When more than one site of metastasis is present, the most advance category is used. pM1c is the most advance.

Value	Value Description
pM0	No distant metastasis
pM1	Distant metastasis
pM1a	Non-regional lymph node(s)
pM1b	Bone(s)
pM1c	Other site(s)*
pMX	Distant metastasis cannot be assessed

CLINICAL STAGING:

112. cT Stage

Definition: **Use Manual for Staging of Cancer, 5thEd. Report cT stage at initial diagnosis.**
Data Type: Character String
Table: tbl_CPCTR.str_240_Clinical_T_Stage
Validation
Rules:
Comments:
1) T0 cases are eligible only for node positive cases where the nodal specimen or metastatic specimen is available and accessioned into the CPCTR.
2) If both clinical and pathologic staging are available, then use pathology.
3) For comments on values below:

*Tumor found in one or both lobes by needle biopsy, but not palpable or reliably visible by imaging, is classified as T1c.
**Invasion into the prostatic apex or into (but not beyond) the prostatic capsule is not classified as T3, but as T2.

Value	Value Description
T0	No evidence of primary tumor
T1	Clinically inapparent tumor not palpable nor visible by imaging
T1a	Tumor incidental histologic finding in 5% or less of tissue resected
T1b	Tumor incidental histologic finding in more than 5% of tissue resected
T1c	Tumor identified by needle biopsy (e.g., because of elevated PSA)
T2	Tumor confined within prostate*
T2a	Tumor involves 1 lobe
T2b	Tumor involves both lobes
T3	Tumor extends through the prostatic capsule**
T3a	Extracapsular extension (unilateral or bilateral)
T3b	Tumor invades seminal vesicle(s)
T4	Tumor is fixed or invades adjacent structures other than seminal vesicles
TX	Primary tumor cannot be assessed

113. cN Stage

Definition: **Use Manual for Staging of Cancer, 5thEd. Report cN stage at initial diagnosis.**
Data Type: Character String
Table: tbl_CPCTR.str_241_Clinical_N_Stage
Validation Rules:
Comments: If used this should be defined by a CT or MRI scan (usually of the pelvis).

Value	Value Description
N0	No regional lymph node metastasis
N1	Metastasis in regional lymph node or nodes
NX	Regional lymph nodes cannot be assessed

114. cM Stage

Definition: **Use Manual for Staging of Cancer, 5thEd. Report cM stage at initial diagnosis.**
Data Type: Character String
Table: tbl_CPCTR.str_242_Clinical_M_Stage
Validation Rules:
Comments:
1) If used this should be defined by either a bone scan (most common), or a CT/MRI of the chest.
2) For comments on values:

*When more than one site of metastasis is present, the most advance category is used. pM1c is the most advance.

Value	Value Description

M0	No distant metastasis
M1	Distant metastasis
M1a	Non-regional lymph node(s)
M1b	Bone(s)
M1c	Other site(s)*
MX	Distant metastasis cannot be assessed

115. General Staging Comments

Definition: **General Comments related to Staging (Pathological and Clinical).**
Data Type: Character String
Table: tbl_CPCTR.str_24_Staging_Comment
Validation Rules:
Comments:

Value	Value Description
Free Text	Open text field (250 alphanumeric characters)

VITAL STATUS/FOLLOW UP DATE

Record the patient's vital status and most recent follow up date.

Vital Status/Follow Up:

116. Date Last Known Alive*:

Definition: **Date should be linked to when the last follow up was done.**
Data Type: Integer: MMYYYY
Table: tbl_CPCTR.int_260_Month_Last_Known_Alive; tbl_CPCTR.int_261_Year_Last_Known_Alive
Validation Rules:
Comments: Record the most recent date on which contact was made with the patient, or it was verified through a tumor registrar, physician, or clinical record that the patient was alive. If the patient has died, then the date in this field remains fixed at the last time the patient was verified to be alive, and no further updating is necessary. The death date (if known) is then entered into Field *Date of Death*. If an attempt was made to follow up the patient history but no new data was collected since the last follow up, make a note in the comments that an attempt was made on a specific date, and that the case may be "lost to follow up".

Value	Value Description
1-12, 00	2-digit month-Month (MM); 00 is standard fictionalized value.

YYYY	4-digit year-Year (YYYY); Must enter a valid year.

117. Date of Death:

Definition: **Enter the date when patient is known to have died.**
Data Type: Integer: MMYYYY
Table: tbl_CPCTR.int_262_Month_of_Death; tbl_CPCTR.int_263_Year_of_Death
Validation Rules:
Comments: If it is known that the patient is dead, but date of death is unknown, code as 00 for month and estimate the year based on medical record (i.e. Enter the year patient was last known alive date). If year but not month is known, then code as 00YYYY.

Value	Value Description
1-12, 00	2-digit month-Month (MM); 00 is standard fictionalized value.
YYYY	4-digit year-Year (YYYY); Must enter a valid year.

118. Vital Status*:

Definition: **Using the most accurate information available, which could include the patient's medical chart, a physician chart, another tumor registry, record patient vital status.**
Data Type: Character String
Table: tbl_CPCTR.str_264_Vital_Status
Validation Rules:
1) If Alive or Lost to follow up, then date of death must be blank and Date last known alive must be completed.
2) If dead, then date of death must not be blank.

Comments: If an attempt was made to follow up the patient history but no new data was collected since the last follow up, make a note in the comments that an attempt was made on a specific date, and that the case may be "lost to follow up".

Value	Value Description
Alive	Alive
Dead	Dead
Dead with warm autopsy	Dead with warm autopsy
Lost to follow up	Must try to find updated follow up information for at least 3 times (once per year). If no new information added, code this.

119. Final Comments:

Definition: **General final comments related to this patient.**
Data Type: Character String
Table: tbl_CPCTR.str_25_Final_Comment
Validation Rules:
Comments: This comments field may be used to describe any information

regarding this index prostate cancer case, including vital status comments.

Value	Value Description
Free Text	Open text field (250 alphanumeric characters)

THERAPY MATRIX

Record the patient's treatment history.

Therapy Matrix: (Items #120-123 are multiple entry fields):

120. Therapy matrix: Type of Therapy

Definition: **Report type of therapy patient was given.**
Data Type: Character String
Table: tbl_Therapy_Matrix.str_Tx_Type
Validation Rules:
Comments: If there is no mention or record of therapy, leave blank, do not assume none was given.

Value	Value Description
Alternative Therapy	Alternative Therapy (specify in comments)
Brachytherapy	Brachytherapy
Chemotherapy	Chemotherapy
Cryotherapy	Cryotherapy
Experimental	Experimental (specify in comments)
External Beam Radiation	External Beam Radiation
Hormone Therapy, NOS	Hormone therapy, NOS
Immunotherapy	Immunotherapy
Medical hormone suppression	Medical hormone suppression
Radiation Therapy, NOS	Radiation Therapy, NOS
Surgical orchiectomy	Surgical orchiectomy
Watchful waiting	Watchful waiting

121. Therapy matrix: Therapy Start Date

Definition: **Enter the date when patient started treatment.**
Data Type: Integer: MMYYYY
Table: tbl_Therapy_Matrix.int_TX_Start_Month; tbl_Therapy_Matrix.int_TX_Start_Year
Validation Rules:
Comments: Include even if therapy is delivered greater than 6 months post diagnosis.

Value	Value Description
1-12, 00	2-digit month-Month (MM); 00 is standard fictionalized value.
YYYY	4-digit year-Year (YYYY); Must enter a valid year.

122. Therapy matrix: Per Initial Treatment Plan? **

Definition: **Report if this treatment was part of the initial, planned therapy.**
Data Type: Character String
Table: tbl_Therapy_Matrix.str_TX_Per_Initial_Treatment_Plan
Validation Rules: If there is no treatment plan, established protocol, or management guidelines, and consultation with a physician advisor is not possible, use the principle: 'initial treatment must begin within four months of the date of initial diagnosis.
Comments:

Value	Value Description
No	No
Yes	Yes
Unknown	Unknown

123. Therapy Matrix Comments:

Definition: **General comments related to this patients treatment.**
Data Type: Character String
Table: tbl_Therapy_Matrix.str_TX_Comment
Validation Rules:
Comments:

Value	Value Description
Free Text	Open text field (250 alphanumeric characters)

Additional overall Therapy comments:

124. Comments on Therapy Response:

Definition: **General comments related to this patients response to treatment.**
Data Type: Character String
Table: tbl_CPCTR.str_276_Therapy_Response_Comment_Field
Validation Rules:
Comments:

Value	Value Description
Free Text	Open text field (250 alphanumeric characters)

125. General Overall Comments on Therapy:

Definition: **General comments related to this patients treatment.**
Data Type: Character String

Table: tbl_CPCTR.str_275_Therapy_Comment_Field
Validation Rules:
Comments:

Value	Value Description
Free Text	Open text field (250 alphanumeric characters)

NEEDLE BIOPSY BLOCK MATRIX: (Items #126-134 are multiple entry fields. Up to 5 blocks preferred)

- **The needle biopsy block matrix section will allow the collection of block details on biopsy samples that meet the minimum requirements for inclusion into the Resource.**
- **For needle biopsy cases, the concern was to give the reviewers some guidance on how many blocks to submit and to clarify what happens when there is only one block with tumor. In these cases it was recommended that the institution cut 3 to 5 blanks for their own diagnostic purposes and then make the residual material available to the CPCTR archive.**
- **We decided that a matrix would be set up and leave the number of blocks submitted to the reviewing pathologist, encouraging them to submit as many blocks as possible on a needle biopsy to a maximum of 5. There is no particular order for entering blocks into the needle biopsy matrix.**
- **Here are the recommended criteria for needle biopsy:**

3) Can include one block or more (up to 5)

4) Must at least include one neoplastic block and classify according to the needle biopsy matrix.
NOTE: for biopsy-only cases, it is a requirement to enter the "subsequent prostatectomy" field in the biopsy attribute section. "Biopsy-Only" cases are cases due to advance disease or other reasons that did NOT lead to a prostatectomy.

126. Biopsy Matrix: Block number

Definition: **Block number of the block with largest tumor nodule.**
Data Type: Character String
Table: tbl_Biopsy_Matrix.str_MB_Block_Number
Validation Rules:
Comments:

Value	Value Description
Free text	List surgical pathology block # from case (varies by institution)

127. Biopsy Matrix: Most Prominent Histological Type of Invasive Cancer

Definition: **Most prominent histological type present on block.**
Data Type: Character String
Table: tbl_Biopsy_Matrix.str_MB_Most_Prominent_Histologic_Type_Invasive_Cancer
Validation Rules: If PIN only, then (*Biopsy-Gleason Grading*) should be blank and (*Biopsy-PIN*) should be checked YES.
Comments:

**Value**	**Value Description**
Adenocarcinoma NOS (aka acinar)	Adenocarcinoma NOS (aka acinar)
Basal cell carcinoma	Basal cell carcinoma
Ductal adenocarcinoma	Ductal adenocarcinoma
Lymphoma	Lymphoma
Mesenchymal tumor (NOS)	Mesenchymal tumor (NOS)
Mucinous adenocarcinoma	Mucinous adenocarcinoma
Neuroendocrine carcinoma	Neuroendocrine carcinoma
PIN only	PIN only
Sarcomatoid carcinoma	Sarcomatoid carcinoma
Signet ring adenocarcinoma	Signet ring adenocarcinoma
Small cell anaplastic carcinoma	Small cell anaplastic carcinoma
Squamous or adenosquamous carcinoma	Squamous or adenosquamous carcinoma
Transitional (not primary)	Transitional (not primary)
Undifferentiated non-small cell carcinoma	Undifferentiated non-small cell carcinoma
Other (specify in comments)	Other (specify in comments)
Unknown	Unknown

128. Biopsy Matrix: Primary Gleason Grade

Definition: **Primary Gleason grade of block**
Data Type: Character String
Table: tbl_Biopsy_Matrix.str_MB_Primary_Gleason_Grade
Validation Rules: If “not adenocarcinoma” then (*Biopsy-Most prominent histological type*) must be either basal cell carcinoma, transitional (not primary), undifferentiated non-small cell carcinoma, sarcomatoid carcinoma, neuroendocrine carcinoma, small cell anaplastic carcinoma, squamous or adenosquamous carcinoma, mesenchymal tumor (NOS), lymphoma, other (specify in comments), or unknown.
Comments:

**Value**	**Value Description**
1	1
2	2
3	3
4	4
5	5
Not adenocarcinoma	Not adenocarcinoma
Not primary tumor	Not primary tumor
PIN only	PIN only
Unknown	Unknown

129. Biopsy Matrix: Secondary Gleason Grade

Definition: **Secondary Gleason grade of block**
Data Type: Character String
Table: tbl_Biopsy_Matrix.str_MB_Secondary_Gleason_Grade
Validation Rules: If "not adenocarcinoma" then (*Biopsy-Most prominent histological type*) must be either basal cell carcinoma, transitional (not primary), undifferentiated non-small cell carcinoma, sarcomatoid carcinoma, neuroendocrine carcinoma, small cell anaplastic carcinoma, squamous or adenosquamous carcinoma, mesenchymal tumor (NOS), lymphoma, other (specify in comments), or unknown.
Comments:

Value	Value Description
1	1
2	2
3	3
4	4
5	5
Not adenocarcinoma	Not adenocarcinoma
Not primary tumor	Not primary tumor
PIN only	PIN only
Unknown	Unknown

130. Biopsy Matrix: Size of Largest Individual Nodule of Invasive Cancer (in cm)

Definition: **Size of invasive cancer present in block.**
Data Type: Double (number)
Table: tbl_Biopsy_Matrix.dbl_MB_Length_of_Core
Validation Rules: Enter a positive number. Enter -1 if unknown.
Comments:

Value	Value Description
Number	-1 Or (>=0 And <100) Or is Null

131. Biopsy Matrix: Presence of High Grade Prostatic Intraepithelial Neoplasia (PIN)

Definition: **Presence of High Grade PIN in block.**
Data Type: Yes/No
Table: tbl_Biopsy_Matrix.str_MB_PIN
Validation Rules:
Comments:

Value	Value Description
Check box	If box is checked, then the PIN value is Yes, otherwise, value is No.

132. Biopsy Matrix: Presence of Perineural Invasion (PN)

Definition: **Presence of Perineural Invasion in block.**
Data Type: Yes/No
Table: tbl_Biopsy_Matrix.str_MB_PN
Validation Rules:
Comments:

Value	Value Description
Check box	If box is checked, then the PN value is Yes, otherwise, value is No.

133. Biopsy Matrix: Presence of Angiolymphatic Invasion (AL)

Definition: **Presence of Angiolymphatic Invasion in block.**
Data Type: Yes/No
Table: tbl_Biopsy_Matrix.str_MB_AL
Validation Rules:
Comments:

Value	Value Description
Check box	If box is checked, then the AL value is Yes, otherwise, value is No.

134. Biopsy Matrix: block comments

Definition: **Comments related to paraffin block matrix.**
Data Type: Character String
Table: tbl_Biopsy_Matrix.str_MB_Comment

Validation Rules:
Comments:

Value	Value Description
Free Text	Open text field (250 alphanumeric characters)

NEEDLE BIOPSY ATTRIBUTES:

- **Enter the overall characteristics of the biopsy.**
- **For "biopsy-only" cases, the subsequent prostatectomy field is a required field.**

NOTE: "Biopsy-Only" cases are cases due to advance disease or other reasons that did NOT lead to a prostatectomy.

135. Biopsy: Most Prominent Histological Type of Invasive Cancer

Definition: **Most prominent overall histological type present in the biopsy.**

Data Type: Character String
Table: tbl_CPCTR.str_Biopsy_Most_Prominent_Histologic_Type
Validation Rules: If PIN only, then (*Biopsy Gleason Grading*) should be "PIN only" and (*Biopsy High Grade PIN*) cannot be "no" or "unknown".
Comments:

**Value**	**Value Description**
Adenocarcinoma NOS (aka acinar)	Adenocarcinoma NOS (aka acinar)
Basal cell carcinoma	Basal cell carcinoma
Ductal adenocarcinoma	Ductal adenocarcinoma
Lymphoma	Lymphoma
Mesenchymal tumor (NOS)	Mesenchymal tumor (NOS)
Mucinous adenocarcinoma	Mucinous adenocarcinoma
Neuroendocrine carcinoma	Neuroendocrine carcinoma
PIN only	PIN only
Sarcomatoid carcinoma	Sarcomatoid carcinoma
Signet ring adenocarcinoma	Signet ring adenocarcinoma
Small cell anaplastic carcinoma	Small cell anaplastic carcinoma
Squamous or adenosquamous carcinoma	Squamous or adenosquamous carcinoma
Transitional (not primary)	Transitional (not primary)
Undifferentiated non-small cell carcinoma	Undifferentiated non-small cell carcinoma
Other (specify in comments)	Other (specify in comments)
Unknown	Unknown

136. Biopsy: Primary Gleason Grade

Definition: **Primary Gleason grade of the biopsy.**
Data Type: Character String
Table: tbl_CPCTR.str_Biopsy_Gleason_Primary_Grade
Validation Rules: If not "adenocarcinoma" then (*Biopsy Most prominent histological type*) must be either basal cell carcinoma, transitional (not primary), undifferentiated non-small cell carcinoma, sarcomatoid carcinoma, neuroendocrine carcinoma, small cell anaplastic carcinoma, squamous or adenosquamous carcinoma, mesenchymal tumor (NOS), lymphoma, other (specify in comments), or unknown.
Comments:

**Value**	**Value Description**
1	1
2	2
3	3
4	4
5	5
Not adenocarcinoma	Not adenocarcinoma
Not primary tumor	Not primary tumor
PIN only	PIN only
Unknown	Unknown

137. Biopsy: Secondary Gleason Grade

Definition: **Secondary Gleason grade of the biopsy.**
Data Type: Character String
Table: tbl_CPCTR.str_Biopsy_Gleason_Secondary_Grade
Validation Rules: If not "adenocarcinoma" then (*Biopsy Most prominent histological type*) must be either basal cell carcinoma, transitional (not primary), undifferentiated non-small cell carcinoma, sarcomatoid carcinoma, neuroendocrine carcinoma, small cell anaplastic carcinoma, squamous or adenosquamous carcinoma, mesenchymal tumor (NOS), lymphoma, other (specify in comments), or unknown.
Comments:

**Value**	**Value Description**
1	1
2	2
3	3
4	4
5	5
Not adenocarcinoma	Not adenocarcinoma
Not primary tumor	Not primary tumor
PIN only	PIN only
Unknown	Unknown

138. Biopsy: Gleason Sum Score

Definition: **Gleason sum score of the biopsy.**
Data Type: Character String
Table: tbl_CPCTR.str_Biopsy_Gleason_Sum_Score
Validation Rules:

1) If not "adenocarcinoma" then (*Biopsy-Most prominent histological type*) must be either basal cell carcinoma, transitional (not primary), undifferentiated non-small cell carcinoma, sarcomatoid carcinoma, neuroendocrine carcinoma, small cell anaplastic carcinoma, squamous or adenosquamous carcinoma, mesenchymal tumor (NOS), lymphoma, other (specify in comments), or unknown.
2) If *Biopsy*-Gleason sum is 6 or 7 must answer "*Biopsy-Percentage of Gleason 4/5 grade (in all of 6's and 7's)*".
3) Sum of character values in (*Biopsy-Primary Gleason*) and (*Biopsy-Secondary Gleason*) must equal sum of value in (*Biopsy-Gleason Sum*).

Comments:

**Value**	**Value Description**
2	2
3	3
4	4
5	5
6	6
7	7
8	8

9	9
10	10
Not adenocarcinoma	Not adenocarcinoma
Not primary tumor	Not primary tumor
PIN only	PIN only
Unknown	Unknown

139. Biopsy: Percentage of Gleason 4/5 grade (in all of 6's and 7's)

Definition: **Percentage of Gleason 4/5 Grade present in the prostatectomy with Gleason sum score of 6 and 7. Underscores prognostic significance of tiny component of Gleason 4 and 5 tumor in Gleason 6 and 7 tumors.**

Data Type: Integer (number)

Table: tbl_CPCTR.int_Biopsy_Percentage_Gleason

Validation Rules:
1) If (*Biopsy-Percentage Gleason 4/5*) is filled in as great than 10, then (*Biopsy*-Gleason Sum) must have a character value of 6 or 7.
2) And character value in either (*Biopsy-Primary Gleason*) and (*Biopsy-Secondary Gleason*) or both must include a "Gleason score 4" or a "Gleason score 5" (i.e. choices are 4,4; 4,5; 5,5; 5,4)

Comments:

Value	Value Description
Integer (%)	-1 Or (>=0 And <=100) Or is Null

140. Percentage of Biopsy Occupied by Tumor

Definition: **Percentage of Gland Occupied by Tumor in the prostatectomy.**

Data Type: Character String

Table: tbl_CPCTR.str_Percentage_Biopsy_Occupied_by_Tumor

Validation Rules: Value (Volume?) may be unknown (-1) only if pathologic T Stage is known (not "TX"), or if pathologic M Stage is "M1".

Comments:

Value	Value Description
<5%	<5%
5 to 25%	5 to 25%
>25%	>25%
Unknown	Unknown

141. Biopsy: Presence of High Grade Prostatic Intraepithelial Neoplasia

Definition: **Is High Grade Prostatic Intraepithelial Neoplasia present in the biopsy?**

Data Type: Character String

Table: tbl_CPCTR.str_Biopsy_Prostatic_Intraepithelial_Neoplasia_Present

Validation Rules:

Comments:

Value	Value Description
No	No
Unknown	Unknown
Yes-focality unknown	Yes-focality unknown
Yes-multifocal	Yes-multifocal
Yes-one or two foci away from the tumor	Yes-one or two foci away from the tumor
Yes-one or two foci in region of tumor	Yes-one or two foci in region of tumor

142. Biopsy: Non-Neoplastic Block #1:

Definition: **Block number of non-neoplastic biopsy block #1**
Data Type: Character String
Table: tbl_CPCTR.str_Biopsy_Nonneoplastic_Block
Validation Rules:
Comments: Should be free of PIN or Cancer if possible.

Value	Value Description
Free Text	List surgical pathology block # from case (varies by institution)

143. Subsequent Prostatectomy:**

Definition: **Specifies whether a subsequent prostatectomy occurred. This field will help clarify cases where only biopsy specimens are available.**
Data Type: Character String
Table: tbl_CPCTR.str_Subsequent_Prostatectomy_Status
Validation Rules:
1) If "No", then prostatectomy attributes and matrix fields should be NULL.
2) If "Yes", then the Prostatectomy Date (if known) must be recorded in the (*Date of prostatectomy*).

Comments:
1) This is a required field for biopsy-only cases.
2) Any other information regarding the off-site prostatectomy can be recorded in the (*subsequent prostatectomy comment*) field.
3) NOTE: "Biopsy-Only" cases are cases due to advance disease or other reasons that did NOT lead to a prostatectomy.

Value	Value Description
No	No
Yes	Yes
Unknown	Unknown

144. Subsequent Prostatectomy comments:

Definition: **General comments related to the subsequent prostatectomy for biopsy-only cases.**
Data Type: Character String
Table: tbl_CPCTR.str_Subsequent_Prostatectomy_Status
Validation

Rules:
Comments: This field is NOT EXPORTED to the CPCTR. ONLY THE HOST SITE WILL HAVE ACCESS TO THE "OFF-SITE" INFORMATION, Due to HIPAA concerns. Its purpose is to:

1) Each site may well keep it for internal use to record any information regarding the "off-site" location and other particulars about the prostatectomy for documentation.
2) NOTE: "Biopsy-Only" cases are cases due to advance disease or other reasons that did NOT lead to a prostatectomy.

Value	Value Description
Free Text	Open text field (250 alphanumeric characters)

145. General Comments on Biopsy:

Definition: **General comments related to the biopsy.**
Data Type: Character String
Table: tbl_CPCTR.str_Biopsy_General_Comment
Validation Rules:
Comments:

Value	Value Description
Free Text	Open text field (250 alphanumeric characters)
