# Supplementary material for: The development of common data elements for a multi-institute prostate cancer tissue bank: The Cooperative Prostate Cancer Tissue Resource (CPCTR) experience
Source: BMC Cancer. 2005 Aug 21;5:108. doi: 10.1186/1471-2407-5-108 (PMC1236914; doi:10.1186/1471-2407-5-108)
Supplement: Additional file 2 — CPCTR CDE paper forms. CPCTR paper forms used for data collection of CDEs by data managers. These forms are used for collecting data at remote locations (i.e. physician offices) and then entered into the database. [file 1471-2407-5-108-S2.pdf]

CPCTR Common Data Elements for Collection of Prostate Cancer Specimens PAPER FORMS version 16 [Data Dictionary Version 22 (09/14/04)]

USER INSTRUCTIONS

Form Completion Guidelines:

1) Priorities for completing the form are to fill out all required elements followed by any subset of data that will allow for inclusion in the CPCTR databank. In our recent conference call there were two approaches to this suggested: **a) fill out the pathology data (suggested by Jan Orenstein) and fill in the clinical sections later, or b) fill in the basic required elements as well as a core of pathology data (Jules Berman, Mike Becich).** In either case, the final goal is to complete as many data elements as possible.
2) Any corrections of changes to the forms should be e-mailed to Ashokkumar A. Patel at patelaa@upmc.edu.

STEPS involved in initial screening, inclusion and classification of CPCTR cases

STEP 1 - Pre-screening cases (Research Assistant's Role) - Required Data Elements Pre-Check - these data element searches are recommended before pathologist review. Make sure the case has the following REQUIRED data elements before beginning microscopic examination:

- **At least 2 blocks are available on case that include prostate tumor *or* 1 block for biopsy-only or metastatic cases.**
- **Date of Birth, Race, Date of Diagnosis and Vital Status must be available to qualify a case and should be collected before microscopic exam and central review by the pathologist is initiated.**
- **Also # Nodes Examined/Positive must be available.**

NOTE: If these data elements are not found, make a record of this case this for later discussion. We must determine the exclusion rate and if too high we may need modified "required" (asterisked) data elements.

STEP 2 - Pathologist Exam - the "pre-screened case" is given to pathologist who begins data recording (Pathologist's Role).

Pathologist determines priorities for entering cases in prostatectomy matrix (Pathologist's Role)

- **Priorities for entering cases in prostatectomy neoplastic block matrix**

- These should assist the teams in picking the highest value blocks for the CPCTR archives.
- Since the matrix can include up to 5 blocks here are the recommendations for selection criteria:

1) The first block should include the largest nodule of tumor (as specified by the CDE)

2) The third through fifth blocks should include seminal vesicle invasion, ECE or angiolymphatic invasion (in that order of preference)
3) The second block should include the largest amount of PIN and preferred is an area of PIN that is independent of cancer (if that exists). If the blocks with PIN all also contain cancer that does not exclude them. Try then to pick a block that has the largest amount of PIN.
4) Try to include at least one block of normal prostate if possible if it is not possible to find a completely normal block then include one with minimal amounts of PIN or carcinoma. Seminal vesicle is not considered a good normal block.

STEP 3 - **Clinical Information Collection** (**Research Assistant's Role**) - If possible, fill out the remaining parts of the form and include the time it took to complete this subsection.

STEP 4 - Needle biopsy CDE guidelines (Pathologist's Role). For needle biopsy cases, a matrix similar to the prostatectomy was developed and the criteria are:
1) Can include one block or more (up to a maximum of 5).
2) Must at least include one block and classify according to the needle biopsy matrix.
3) Try to include one block of normal prostate if possible.

STEP 5 - Frozen Tissue Prostatectomy archive guidelines (Pathologist's Role)

- Since we have not really started accruing cases it was decided to keep the exclusionary criteria to a minimum until we have a better idea from each site actually reviewing cases.
- Therefore the only criteria we recommend currently are:
    - At least 1 neoplastic block or more need to be able to be entered into the matrix or case is excluded.

STEP 6 - Metastasis CDE guidelines (Pathologist's Role).
For metastatic cases, a matrix similar to the prostatectomy was developed and the criteria are:
1) Can include one block or more (up to a maximum of 5).
2) Must at least include one block and classify according to the metastatic matrix.
3) Try to include one block of normal metastatic tissue if possible.

Demographics

Internal Reference Data (This section is not to be supplied to CPCTR only for internal record keeping use only and not to be passed on to IMS)

1. **Hospital Identifier:** ____________________________________

2. **Last Name:** ____

3. **First Name:** ____

Demographics

4. Case Identifier*: ____
(10-digit alphanumeric provided by IMS)

5. Race*: **(circle one):** **Caucasian** **Pacific Islander**

African American **Hispanic** **Asian** **Native American**

Unknown **Not Available** **Other (write in if known):___________**

6. Other Race comments:
____
____
____
____

7. Hispanic Origin: **(circle one)** **Yes** **No** **Unknown**

8. Birth date*: __ __ / __ __ __ __ **(MM/YYYY)**

Clinical History

9. Family History of Prostate Cancer:

(circle one) **Yes** **No** **Unknown**

10. Family History comments
____
____
____
____

11. Date of Diagnosis/Date of biopsy proven cancer*:

__ __ / __ __ __ __ **(MM/YYYY)**

12. General Demographic Comment Field:
____
____
____
____

SPECIMEN AVAILABLITY

This section will show the types of specimens available through the Resource.

NOTE: *** Case inclusion criteria: Any ONE question #13-20 below must say "YES".**
(Specific inclusion criteria for each of the bio-specimens below are listed in their respective section)

13. **Are Prostatectomy specimens available?***

    (circle one) Yes No Unknown

14. **Are Biopsy specimens available?***

    (circle one) Yes No Unknown

15. **Are Regional Lymph Node specimens available?***

    (circle one) Yes No Unknown

16. **Are Metastatic specimens available?***

    (circle one) Yes No Unknown

17. **Are Plasma samples available?***

    (circle one) Yes No Unknown

18. **Are Serum samples available?***

    (circle one) Yes No Unknown

19. **Are Red Blood Cells (RBCs) available?***

    (circle one) Yes No Unknown

20. **Are Peripheral Blood Mononuclear Cells (PBMCs) available?***

    (circle one) Yes No Unknown

PROSTATIC SPECIFIC ANTIGEN (PSA)

DIAGNOSTIC PSA:

21. PSA value that prompted Diagnostic Biopsy which showed cancer

    __ __ __ __ . __ [>0.0; 9999.0, -1 (unknown)]

22. Date of PSA that Prompted Diagnostic Biopsy which showed cancer

    __ __ / __ __ __ __ (MM/YYYY)

23. General comments for Diagnostic PSA:

    ____
    ____
    ____
    ____

PSA MATRIX: (Items #24-27 are multiple entry fields)

The PSA matrix section will allow the collection of multiple PSA values that are prior to and after the diagnostic PSA value. We can write scripts to separate the pre-biopsy (for biopsy-only cases)/pre-prostatectomy PSAs from the post-biopsy/pre-prostatectomy PSAs to determine the biochemical recurrence. We can also calculate the diagnostic PSA since we know the biopsy date and all the PSA dates, and will identify the date of the PSA just before the biopsy. Thus, the central database will take the PSA and Date, sort by date, and calculate the needed results. This also allows one to enter in dates in any order (if much later you get an old chart with more values).

24. PSA below threshold (<0.1):
25. PSA value (pre/post diagnostic only)
26. Date of PSA
27. General comments for PSA matrix

(PLEASE ENTER DATA IN THE PSA MATRIX TABLE BELOW)

PSA MATRIX TABLE:

PSA below threshold (<0.1) (Circle YES or NO)	PSA value (pre / post diagnostic only) [>0.0; 9999.0, -1 (unknown)]	Date of PSA (Month) (MM)	Date of PSA (Year) (YYYY)	General comments for PSA matrix
Y N				
Y N				
Y N				
Y N				
Y N				
Y N				
Y N				
Y N				
Y N				
Y N				
Y N				
Y N				
Y N				
Y N				
Y N				

PSA MATRIX TABLE:

PSA below threshold (<0.1) (Circle YES or NO)	PSA value (pre / post diagnostic only) [>0.0; 9999.0, -1 (unknown)]	Date of PSA (Month) (MM)	Date of PSA (Year) (YYYY)	General comments for PSA matrix
Y N				
Y N				
Y N				
Y N				
Y N				
Y N				
Y N				
Y N				
Y N				
Y N				
Y N				
Y N				
Y N				
Y N				
Y N				

ANATOMICAL PATHOLOGY GRADING AND CHARACTERISTICS

PARAFFIN BLOCK MATRIX:(Items #28-40 are multiple entry fields. Up to 5 blocks preferred)

The Paraffin block matrix section will allow the collection of block details on cases that meet the minimum requirements for inclusion (1 paraffin blocks with tumor) into the Resource. NOTE: Priorities for entering cases in prostatectomy neoplastic block matrix

- **These should assist the teams in picking the highest value blocks for the CPCTR archives.**
- **Since the matrix can include up to 5 blocks here are the recommendations for selection criteria:**
  1) **The *first block (block a)* should include the largest nodule of tumor (as specified by the CDE)**
  2) **The *third through fifth blocks (blocks c, d, e)* should include seminal vesicle invasion, ECE or angiolymphatic invasion (in that order of preference) NOTE: If SV, ECE, AL do not occur, select the next largest area of tumor for 3 to 5 of the matrix.**
  3) **The *second block (block b)* should include the largest amount of PIN and preferred is an area of PIN that is independent of cancer (if that exists). If the blocks with PIN all also contain cancer that does not exclude them. Try then to pick a block that has the largest amount of PIN.**

NOTE: This section is for the PARAFFIN BLOCK matrix.

It is very possible that some (and occasionally all) of the paraffin blocks in the matrix will also have frozen tissue. When this happens indicate this in items 39a-e. On occasion, there may be some blocks that are only frozen (site dependent) also indicate this in the aforementioned data items.

==

28a. Paraffin Block Matrix a: Block number**
(Block with largest tumor nodule)
Accession and Block # (i.e. SP-91-645-1A):

__ __ __ - __ __ - __ __ __ __ __ - __ __ __ __

29a. Paraffin Block Matrix: Most Prominent Histological Type of Invasive Cancer ** (Circle One)

Adenocarcinoma NOS (aka acinar)   Undifferentiated non-small cell carcinoma

Mucinous adenocarcinoma   Signet ring adenocarcinoma   Lymphoma

Transitional (not primary)   Ductal adenocarcinoma   Neuroendocrine carcinoma

Small cell anaplastic carcinoma   Sarcomatoid carcinoma   Basal cell carcinoma

Squamous or adenosquamous carcinoma   Mesenchymal tumor (NOS)

PIN only   Unknown   Other (specify in comments)

30a. Paraffin Block Matrix: Primary Gleason Grade (Circle One)**

Gleason grade: 1   2   3   4   5

not adenocarcinoma   not primary tumor   Unknown   PIN only

31a. Paraffin Block Matrix: Secondary Gleason Grade (Circle One)**

Gleason grade: 1   2   3   4   5

not adenocarcinoma   not primary tumor   Unknown   PIN only

32a. Paraffin Block Matrix: Size of Largest Individual Nodule of Invasive Cancer (in cm)
__ __ . __ cm (-1=unknown, 0.0 – 99.9 cm)

(33a-39a) BLOCK ATTRIBUTES: Presence of:
High Grade Prostatic Intraepithelial Neoplasia (PIN); Extracapsular Extension (ECE); Positive Surgical Margin (SM); Perineural Invasion (PN); Seminal Vesicle Invasion (SV); Angiolymphatic Invasion (AL); Type of Block(s) available (circle all that apply)

PIN   ECE   SM   PN   SV   AL   None

Paraffin only   Frozen only   Paraffin and frozen

40a. Paraffin Block Matrix: block comments
____
____

==

28b. Paraffin Block Matrix b: Block number:** **<u>(Block with largest amount of PIN, preferably in area independent of tumor, if possible. If no PIN then sample block with next largest amount of tumor.)</u>**

Accession and Block # (i.e. SP-91-645-1A):

__ __ __ - __ __ - __ __ __ __ __ - __ __ __ __

29b. Paraffin Block Matrix: Most Prominent Histological Type of Invasive Cancer ** (Circle One)

Adenocarcinoma NOS (aka acinar) Undifferentiated non-small cell carcinoma

Mucinous adenocarcinoma Signet ring adenocarcinoma Lymphoma

Transitional (not primary) Ductal adenocarcinoma Neuroendocrine carcinoma

Small cell anaplastic carcinoma Sarcomatoid carcinoma Basal cell carcinoma

Squamous or adenosquamous carcinoma Mesenchymal tumor (NOS)

PIN only Unknown Other (specify in comments)

30b. Paraffin Block Matrix: Primary Gleason Grade (Circle One)**

Gleason grade: 1 2 3 4 5

not adenocarcinoma not primary tumor Unknown PIN only

31b. Paraffin Block Matrix: Secondary Gleason Grade (Circle One)**

Gleason grade: 1 2 3 4 5

not adenocarcinoma not primary tumor Unknown PIN only

32b. Paraffin Block Matrix: Size of Largest Individual Nodule of Invasive Cancer (in cm)

__ __ . __ cm (-1=unknown, 0.0 – 99.9 cm)

(33b-39b) BLOCK ATTRIBUTES: Presence of: High Grade Prostatic Intraepithelial Neoplasia (PIN); Extracapsular Extension (ECE); Positive Surgical Margin (SM); Perineural Invasion (PN); Seminal Vesicle Invasion (SV); Angiolymphatic Invasion (AL); Type of Block(s) available (circle all that apply)

PIN ECE SM PN SV AL None

Paraffin only Frozen only Paraffin and frozen

40b. Paraffin Block Matrix: block comments

____

____

==

28c. Paraffin Block Matrix c: Block number: <u>(Block with seminal vesicle invasion. If no seminal vesicle invasion, include block with next largest tumor nodule).</u>**

Accession and Block # (i.e. SP-91-645-1A):

__ __ __ - __ __ - __ __ __ __ __ - __ __ __ __

29c. Paraffin Block Matrix: Most Prominent Histological Type of Invasive Cancer ** (Circle One)

Adenocarcinoma NOS (aka acinar)   Undifferentiated non-small cell carcinoma

Mucinous adenocarcinoma   Signet ring adenocarcinoma   Lymphoma

Transitional (not primary)   Ductal adenocarcinoma   Neuroendocrine carcinoma

Small cell anaplastic carcinoma   Sarcomatoid carcinoma   Basal cell carcinoma

Squamous or adenosquamous carcinoma   Mesenchymal tumor (NOS)

PIN only   Unknown   Other (specify in comments)

30c. Paraffin Block Matrix: Primary Gleason Grade (Circle One)**

Gleason grade: 1   2   3   4   5

not adenocarcinoma   not primary tumor   Unknown   PIN only

31c. Paraffin Block Matrix: Secondary Gleason Grade (Circle One)**

Gleason grade: 1   2   3   4   5

not adenocarcinoma   not primary tumor   Unknown   PIN only

32c. Paraffin Block Matrix: Size of Largest Individual Nodule of Invasive Cancer (in cm)

__ __ . __ cm (-1=unknown, 0.0 – 99.9 cm)

**(33c-39c) BLOCK ATTRIBUTES: Presence of:
High Grade Prostatic Intraepithelial Neoplasia (PIN); Extracapsular Extension (ECE); Positive Surgical Margin (SM); Perineural Invasion (PN); Seminal Vesicle Invasion (SV); Angiolymphatic Invasion (AL); Type of Block(s) available (circle all that apply)**

PIN   ECE   SM   PN   SV   AL   None

Paraffin only   Frozen only   Paraffin and frozen

40c. Paraffin Block Matrix: block comments

____
____

==

28d. Paraffin Block Matrix d: Block number: <u>(Block with extracapsular extension (ECE) if present. If ECE is not present pick the block with the next largest amount of tumor).</u>**

Accession and Block # (i.e. SP-91-645-1A):

__ __ __ - __ __ - __ __ __ __ __ - __ __ __ __

29d. Paraffin Block Matrix: Most Prominent Histological Type of Invasive Cancer ** (Circle One)

Adenocarcinoma NOS (aka acinar)   Undifferentiated non-small cell carcinoma

Mucinous adenocarcinoma   Signet ring adenocarcinoma   Lymphoma

Transitional (not primary)   Ductal adenocarcinoma   Neuroendocrine carcinoma

Small cell anaplastic carcinoma   Sarcomatoid carcinoma   Basal cell carcinoma

Squamous or adenosquamous carcinoma   Mesenchymal tumor (NOS)

PIN only   Unknown   Other (specify in comments)

30d. Paraffin Block Matrix: Primary Gleason Grade (Circle One)**

Gleason grade: 1   2   3   4   5

not adenocarcinoma   not primary tumor   Unknown   PIN only

31d. Paraffin Block Matrix: Secondary Gleason Grade (Circle One)**

Gleason grade: 1   2   3   4   5

not adenocarcinoma   not primary tumor   Unknown   PIN only

32d. Paraffin Block Matrix: Size of Largest Individual Nodule of Invasive Cancer (in cm)

__ __ . __ cm (-1=unknown, 0.0 – 99.9 cm)

(33d-39d) BLOCK ATTRIBUTES: Presence of:
High Grade Prostatic Intraepithelial Neoplasia (PIN); Extracapsular Extension (ECE); Positive Surgical Margin (SM); Perineural Invasion (PN); Seminal Vesicle Invasion (SV); Angiolymphatic Invasion (AL); Type of Block(s) available (circle all that apply)

PIN   ECE   SM   PN   SV   AL   None

Paraffin only   Frozen only   Paraffin and frozen

40d. Paraffin Block Matrix: block comments

____
____

==

28e. Paraffin Block Matrix e: Block number: <u>(Block with angiolymphatic invasion (AI). If AI is not present pick the block with the next largest amount of tumor).</u>**

Accession and Block # (i.e. SP-91-645-1A):

__ __ __ - __ __ - __ __ __ __ __ - __ __ __ __

29e. Paraffin Block Matrix: Most Prominent Histological Type of Invasive Cancer ** (Circle One)

Adenocarcinoma NOS (aka acinar) Undifferentiated non-small cell carcinoma

Mucinous adenocarcinoma Signet ring adenocarcinoma Lymphoma

Transitional (not primary) Ductal adenocarcinoma Neuroendocrine carcinoma

Small cell anaplastic carcinoma Sarcomatoid carcinoma Basal cell carcinoma

Squamous or adenosquamous carcinoma Mesenchymal tumor (NOS)

PIN only Unknown Other (specify in comments)

30e. Paraffin Block Matrix: Primary Gleason Grade (Circle One)**

Gleason grade: 1 2 3 4 5

not adenocarcinoma not primary tumor Unknown PIN only

31e. Paraffin Block Matrix: Secondary Gleason Grade (Circle One)**

Gleason grade: 1 2 3 4 5

not adenocarcinoma not primary tumor Unknown PIN only

32e. Paraffin Block Matrix: Size of Largest Individual Nodule of Invasive Cancer (in cm)

__ __ . __ cm (-1=unknown, 0.0 – 99.9 cm)

(33e-39e) BLOCK ATTRIBUTES: Presence of: High Grade Prostatic Intraepithelial Neoplasia (PIN); Extracapsular Extension (ECE); Positive Surgical Margin (SM); Perineural Invasion (PN); Seminal Vesicle Invasion (SV); Angiolymphatic Invasion (AL); Type of Block(s) available (circle all that apply)

PIN ECE SM PN SV AL None

Paraffin only Frozen only Paraffin and frozen

40e. Paraffin Block Matrix: block comments

____
____

NON-NEOPLASTIC PARAFFIN BLOCK'S:

Try to include at least one block of normal prostate if possible (two blocks are preferred). If it is not possible to find a completely normal block then include one with minimal amounts of PIN or carcinoma. Seminal vesical is not considered a good normal block.

41.Paraffin Prostatectomy Non-Neoplastic Block #1:

Accession and Block # (i.e. SP-91-645-1A):

__ __ __ - __ __ - __ __ __ __ __ - __ __ __ __

42.Paraffin Prostatectomy Non-Neoplastic Block #2:

Accession and Block # (i.e. SP-91-645-1A):

__ __ __ - __ __ - __ __ __ __ __ - __ __ __ __

43.General Comments on Paraffin Blocks:

____
____
____
____

FROZEN BLOCK MATRIX: (Items #44-56 are multiple entry fields. Up to 5 blocks preferred)

NOTE: This section is for the FROZEN BLOCK matrix.
It is very possible that some (and occasionally all) of the frozen blocks in the matrix will also have paraffin tissue. When this happens indicate this in data element [Type of Block(s) available]. On occasion, there may be some blocks that are only frozen (site dependent) also indicate this in the aforementioned data items.

The Frozen block matrix section will allow the collection of block details on cases that meet the minimum requirements for inclusion into the Resource.
NOTE: Priorities for entering cases in prostatectomy neoplastic block matrix

- **These should assist the teams in picking the highest value blocks for the CPCTR archives.**
- **Since the matrix can include up to 5 blocks here are the recommendations for selection criteria:**
  1) **The *first block (block a)* should include the largest nodule of tumor (as specified by the CDE)**
  2) **The *third through fifth blocks (blocks c, d, e)* should include seminal vesicle invasion, ECE or angiolymphatic invasion (in that order of preference) NOTE: If SV, ECE, AL do not occur, select the next largest area of tumor for 3 to 5 of the matrix.**
  3) **The *second block (block b)* should include the largest amount of PIN and preferred is an area of PIN that is independent of cancer (if that exists). If the blocks with PIN all also contain cancer that does not exclude them. Try then to pick a block that has the largest amount of PIN.**

NOTE: This section is for the FROZEN BLOCK matrix.
It is very possible that some (and occasionally all) **of the frozen blocks in the matrix will also have paraffin tissue.** When this happens indicate this in items 55a-e). **On occasion**, there may be some blocks that are only frozen (site dependent) also indicate this in the aforementioned data items.

==

44a. Frozen Block Matrix a: Block number**
(Block with largest tumor nodule)
Accession and Block # (i.e. SP-91-645-1A):

__ __ __ - __ __ - __ __ __ __ __ - __ __ __ __

45a. Frozen Block Matrix: Most Prominent Histological Type of Invasive Cancer ** (Circle One)

Adenocarcinoma NOS (aka acinar)   Undifferentiated non-small cell carcinoma

Mucinous adenocarcinoma   Signet ring adenocarcinoma   Lymphoma

Transitional (not primary)   Ductal adenocarcinoma   Neuroendocrine carcinoma

Small cell anaplastic carcinoma   Sarcomatoid carcinoma   Basal cell carcinoma

Squamous or adenosquamous carcinoma   Mesenchymal tumor (NOS)

PIN only   Unknown   Other (specify in comments)

46a. Frozen Block Matrix: Primary Gleason Grade (Circle One)**

Gleason grade: 1   2   3   4   5

not adenocarcinoma   not primary tumor   Unknown   PIN only

47a. Frozen Block Matrix: Secondary Gleason Grade (Circle One)**

Gleason grade: 1   2   3   4   5

not adenocarcinoma   not primary tumor   Unknown   PIN only

48a. Frozen Block Matrix: Size of Largest Individual Nodule of Invasive Cancer (in cm)
__ __ . __ cm (-1=unknown, 0.0 – 99.9 cm)

(49a-55a) BLOCK ATTRIBUTES: Presence of:
High Grade Prostatic Intraepithelial Neoplasia (PIN); Extracapsular Extension (ECE); Positive Surgical Margin (SM); Perineural Invasion (PN); Seminal Vesicle Invasion (SV); Angiolymphatic Invasion (AL); Type of Block(s) available (circle all that apply)

PIN   ECE   SM   PN   SV   AL   None

Paraffin only   Frozen only   Paraffin and frozen

56a. Frozen Block Matrix: block comments

____
____

==

44b. Frozen Block Matrix b: Block number: <u>(Block with largest amount of PIN, preferably in area independent of tumor, if possible. If no PIN then sample block with next largest amount of tumor.)</u>**

Accession and Block # (i.e. SP-91-645-1A):

__ __ __ - __ __ - __ __ __ __ __ - __ __ __ __

45b. Frozen Block Matrix: Most Prominent Histological Type of Invasive Cancer ** (Circle One)

Adenocarcinoma NOS (aka acinar)   Undifferentiated non-small cell carcinoma

Mucinous adenocarcinoma   Signet ring adenocarcinoma   Lymphoma

Transitional (not primary)   Ductal adenocarcinoma   Neuroendocrine carcinoma

Small cell anaplastic carcinoma   Sarcomatoid carcinoma   Basal cell carcinoma

Squamous or adenosquamous carcinoma   Mesenchymal tumor (NOS)

PIN only   Unknown   Other (specify in comments)

46b. Frozen Block Matrix: Primary Gleason Grade (Circle One)**

Gleason grade: 1   2   3   4   5

not adenocarcinoma   not primary tumor   Unknown   PIN only

47b. Frozen Block Matrix: Secondary Gleason Grade (Circle One)**

Gleason grade: 1   2   3   4   5

not adenocarcinoma   not primary tumor   Unknown   PIN only

48b. Frozen Block Matrix: Size of Largest Individual Nodule of Invasive Cancer (in cm)

__ __ . __ cm (-1=unknown, 0.0 – 99.9 cm)

(49b-55b) BLOCK ATTRIBUTES: Presence of:
High Grade Prostatic Intraepithelial Neoplasia (PIN); Extracapsular Extension (ECE); Positive Surgical Margin (SM); Perineural Invasion (PN); Seminal Vesicle Invasion (SV); Angiolymphatic Invasion (AL); Type of Block(s) available (circle all that apply)

PIN   ECE   SM   PN   SV   AL   None

Paraffin only   Frozen only   Paraffin and frozen

56b. Frozen Block Matrix: block comments

____
____
==

44c. Frozen Block Matrix c: Block number: <u>(Block with seminal vesicle invasion. If no seminal vesicle invasion, include block with next largest tumor nodule).</u>**

Accession and Block # (i.e. SP-91-645-1A):

__ __ __ - __ __ - __ __ __ __ __ - __ __ __ __

45c. Frozen Block Matrix: Most Prominent Histological Type of Invasive Cancer ** (Circle One)

Adenocarcinoma NOS (aka acinar)   Undifferentiated non-small cell carcinoma

Mucinous adenocarcinoma   Signet ring adenocarcinoma   Lymphoma

Transitional (not primary)   Ductal adenocarcinoma   Neuroendocrine carcinoma

Small cell anaplastic carcinoma   Sarcomatoid carcinoma   Basal cell carcinoma

Squamous or adenosquamous carcinoma   Mesenchymal tumor (NOS)

PIN only   Unknown   Other (specify in comments)

46c. Frozen Block Matrix: Primary Gleason Grade (Circle One)**

Gleason grade: 1   2   3   4   5

not adenocarcinoma   not primary tumor   Unknown   PIN only

47c. Frozen Block Matrix: Secondary Gleason Grade (Circle One)**

Gleason grade: 1   2   3   4   5

not adenocarcinoma   not primary tumor   Unknown   PIN only

48c. Frozen Block Matrix: Size of Largest Individual Nodule of Invasive Cancer (in cm)

__ __ . __ cm (-1=unknown, 0.0 – 99.9 cm)

(49c-55c) BLOCK ATTRIBUTES: Presence of: High Grade Prostatic Intraepithelial Neoplasia (PIN); Extracapsular Extension (ECE); Positive Surgical Margin (SM); Perineural Invasion (PN); Seminal Vesicle Invasion (SV); Angiolymphatic Invasion (AL); Type of Block(s) available (circle all that apply)

PIN   ECE   SM   PN   SV   AL   None

Paraffin only   Frozen only   Paraffin and frozen

56c. Frozen Block Matrix: block comments

____

____

==

44d. Frozen Block Matrix d: Block number: <u>(Block with extracapsular extension (ECE) if present. If ECE is not present pick the block with the next largest amount of tumor).</u>**

Accession and Block # (i.e. SP-91-645-1A):

__ __ __ - __ __ - __ __ __ __ __ - __ __ __ __

45d. Frozen Block Matrix: Most Prominent Histological Type of Invasive Cancer ** (Circle One)

Adenocarcinoma NOS (aka acinar) Undifferentiated non-small cell carcinoma

Mucinous adenocarcinoma Signet ring adenocarcinoma Lymphoma

Transitional (not primary) Ductal adenocarcinoma Neuroendocrine carcinoma

Small cell anaplastic carcinoma Sarcomatoid carcinoma Basal cell carcinoma

Squamous or adenosquamous carcinoma Mesenchymal tumor (NOS)

PIN only Unknown Other (specify in comments)

46d. Frozen Block Matrix: Primary Gleason Grade (Circle One)**

Gleason grade: 1 2 3 4 5

not adenocarcinoma not primary tumor Unknown PIN only

47d. Frozen Block Matrix: Secondary Gleason Grade (Circle One)**

Gleason grade: 1 2 3 4 5

not adenocarcinoma not primary tumor Unknown PIN only

48d. Frozen Block Matrix: Size of Largest Individual Nodule of Invasive Cancer (in cm)

__ __ . __ cm (-1=unknown, 0.0 – 99.9 cm)

(49d-55d) BLOCK ATTRIBUTES: Presence of: High Grade Prostatic Intraepithelial Neoplasia (PIN); Extracapsular Extension (ECE); Positive Surgical Margin (SM); Perineural Invasion (PN); Seminal Vesicle Invasion (SV); Angiolymphatic Invasion (AL); Type of Block(s) available (circle all that apply)

PIN ECE SM PN SV AL None

Paraffin only Frozen only Paraffin and frozen

56d. Frozen Block Matrix: block comments

____
____

==

44e. Frozen Block Matrix e: Block number: <u>(Block with angiolymphatic invasion (AI). If AI is not present pick the block with the next largest amount of tumor).</u>**

Accession and Block # (i.e. SP-91-645-1A):

__ __ __ - __ __ - __ __ __ __ __ - __ __ __ __

45e. Frozen Block Matrix: Most Prominent Histological Type of Invasive Cancer ** (Circle One)

Adenocarcinoma NOS (aka acinar)   Undifferentiated non-small cell carcinoma

Mucinous adenocarcinoma   Signet ring adenocarcinoma   Lymphoma

Transitional (not primary)   Ductal adenocarcinoma   Neuroendocrine carcinoma

Small cell anaplastic carcinoma   Sarcomatoid carcinoma   Basal cell carcinoma

Squamous or adenosquamous carcinoma   Mesenchymal tumor (NOS)

PIN only   Unknown   Other (specify in comments)

46e. Frozen Block Matrix: Primary Gleason Grade (Circle One)**

Gleason grade: 1   2   3   4   5

not adenocarcinoma   not primary tumor   Unknown   PIN only

47e. Frozen Block Matrix: Secondary Gleason Grade (Circle One)**

Gleason grade: 1   2   3   4   5

not adenocarcinoma   not primary tumor   Unknown   PIN only

48e. Frozen Block Matrix: Size of Largest Individual Nodule of Invasive Cancer (in cm)

__ __ . __ cm (-1=unknown, 0.0 – 99.9 cm)

(49e-55e) BLOCK ATTRIBUTES: Presence of:
High Grade Prostatic Intraepithelial Neoplasia (PIN); Extracapsular Extension (ECE); Positive Surgical Margin (SM); Perineural Invasion (PN); Seminal Vesicle Invasion (SV); Angiolymphatic Invasion (AL); Type of Block(s) available (circle all that apply)

PIN   ECE   SM   PN   SV   AL   None

Paraffin only   Frozen only   Paraffin and frozen

56e. Frozen Block Matrix: block comments

____
____

NON-NEOPLASTIC FROZEN BLOCK'S:

Try to include at least one block of normal prostate if possible (two blocks are preferred). If it is not possible to find a completely normal block then include one with minimal amounts of PIN or carcinoma. Seminal vesical is not considered a good normal block.

57.Frozen Prostatectomy Non-Neoplastic Block #1:
Accession and Block # (i.e. SP-91-645-1A):

__ __ __ - __ __ - __ __ __ __ __ - __ __ __ __

58.Frozen Prostatectomy Non-Neoplastic Block #2:
Accession and Block # (i.e. SP-91-645-1A):

__ __ __ - __ __ - __ __ __ __ __ - __ __ __ __

59.Frozen Tissue: Warm Ischemic Time (in minutes)**

__ __ __ __ __ minutes (0 to 99; use –1 for unknown)

60.General Comments on Frozen Blocks:

____
____
____
____

HISTOLOGICAL CHARACTERISTICS OF PROSTATECTOMY

Enter the overall characteristics of the prostatectomy.

61.Date of Prostatectomy**

__ __ / __ __ __ __ **(MM/YYYY)**

62.Is Residual Carcinoma Present at Prostatectomy?**

(circle one)

Yes **No** **HGPIN only** **Donor** **Unknown**

63.Lobe Laterality

(circle one) **One - unspecified (laterality unknown)**

One – right lobe only **One – left lobe only**

Two – bilateral involvement

64.Most Prominent Histological Type of Invasive Cancer** (Circle One)

Adenocarcinoma NOS (aka acinar) Undifferentiated non-small cell carcinoma

Mucinous adenocarcinoma Signet ring adenocarcinoma Lymphoma

Transitional (not primary) Ductal adenocarcinoma Neuroendocrine carcinoma

Small cell anaplastic carcinoma Sarcomatoid carcinoma Basal cell carcinoma

Squamous or adenosquamous carcinoma Mesenchymal tumor (NOS)

PIN only Unknown Other (specify in comments)

Comments:____
____
____

65.2nd Most Prominent Histological Type of Invasive Cancer (Circle One)

Adenocarcinoma NOS (aka acinar) Undifferentiated non-small cell carcinoma

Mucinous adenocarcinoma Signet ring adenocarcinoma Lymphoma

Transitional (not primary) Ductal adenocarcinoma Neuroendocrine carcinoma

Small cell anaplastic carcinoma Sarcomatoid carcinoma Basal cell carcinoma

Squamous or adenosquamous carcinoma Mesenchymal tumor (NOS)

PIN only Unknown Other (specify in comments)

Comments:____
____
____

66.Prostatectomy: Primary Gleason Grade** (Circle One)

Gleason grade: 1 2 3 4 5

not adenocarcinoma not primary tumor Unknown PIN only

67.Prostatectomy: Secondary Gleason Grade** (Circle One)

Gleason grade: 1 2 3 4 5

not adenocarcinoma not primary tumor Unknown PIN only

68.Prostatectomy: Gleason Sum Score** (Circle One)

Gleason score 2	Gleason score 3	Gleason score 4
Gleason score 5	Gleason score 6	Gleason score 7
Gleason score 8	Gleason score 9	Gleason score 10
not adenocarcinoma	not primary tumor	unknown

69.Percentage of Gleason 4/5 grade (in all of 6's and 7's):

__ __ __ % (0.x – 100 %, -1 = unknown)

70.Size of Largest Individual Nodule of Invasive Cancer (in cm):

__ __ __ . __ cm (0.x – 999, -1 = unknown)

71.Percentage of Gland Occupied by Tumor: (Circle One)

<5% 5 to 25% >25% Unknown

72.Multifocal Disease: (Circle One)

Definition: **Multifocal tumors MUST be separated by a certain distance (1-2 cm) so that the chance of artifactual sectioning is eliminated. The INDEX tumor is the primary case and the size is determined from that tumor.**

Yes No Unknown

73.Presence of High Grade Prostatic Intraepithelial Neoplasia: (Circle One)

Yes - # of foci unknown	Yes – 1 or 2 foci in region of tumor
Yes – 1 or 2 foci away from tumor	Yes – multifocal
No	Unknown

74. Extraprostatic Extension / Extracapsular Invasion: (Circle One)

Definition: **Extraprostatic or Extracapsular extension seen in prostatectomy. Focal Extracapsular extension is defined as less than 0.8mm of ECE and established is >0.8mm. Multifocal is greater than 2 different foci of ECE.**

None **Focal** **Multifocal** **Established** **Unknown**

75. Surgical Margin Involvement: (Circle One)

All surgical margins free of tumor **Tumor focal at margin**

Tumor widespread at margin **Unknown**

76. Presence of Perineural Invasion: (Circle One)

Yes **No** **Unknown**

77. Presence of Seminal Vesicle Invasion: (Circle One)

Yes **No** **Unknown**

78. Presence of Angiolymphatic Invasion: (Circle One)

Yes **No** **Unknown**

79. General Comments Section for Prostatectomy:

____
____
____
____

REGIONAL LYMPH NODE STATUS AT THE TIME OF PROSTATECTOMY

Include the lymph node status *at the time of the prostatectomy*.
-If there is regional lymph node exploration prior or equal to the prostatectomy date, but after the initial diagnostic biopsy, then enter values here (in the Lymph Node section).
-Regional lymph nodes removed after the prostatectomy or Distant lymph nodes should be enter in the *METASTASIS MATRIX* section.
NOTE:
***Regional lymph nodes* are of the true pelvis. They are pelvic nodes below the bifurcation of the common iliac arteries and include: Pelvic (NOS), Hypogastric, Obturator, Iliac (internal, external, NOS), and Sacral (lateral, presacral, promontory, NOS) nodes.**
***Distant lymph nodes* are outside the confines of the true pelvis and their involvement constitutes distant metastasis. They can be imaged using ultrasound, computed tomography, magnetic resonance imaging, or lymphangiography, and include: aortic (para-aortic, periaortic, lumbar), common iliac, inguinal, superficial inguinal (femoral), supraclavicular, cervical, scalene, and retroperitoneal (NOS) nodes.**

80.Date of Regional Lymph Node resection

__ __ / __ __ __ __ **(MM/YYYY)**

81.Nodes Examined**

Comments:
1) NOTE: -1 = “nodes examined, but number unknown”
2) If no nodes are examined or do not know if any were examined, then enter “0”.

# of examined nodes:__ __ (> 0; 99, -1 = “nodes examined, number unknown”)

82.Nodes Positive**

Comments:
1) NOTE: -1 = “*some nodes are positive, but number unknown*”
2) If no nodes are positive or do not know if any were positive, then enter “0”.

# of positive nodes: __ __ (> 0; 99, -1 = “nodes examined, number unknown”)

83.Lymph Node Non-Neoplastic Block #1:

Accession and Block # (i.e. SP-91-645-1A):

__ __ __ - __ __ - __ __ __ __ __ - __ __ __ __

84.General Comments Section for Lymph Nodes:

____
____
____
____

REGIONAL LYMPH NODE BLOCK MATRIX: [Items #85-87 are multiple entry fields. <u>Up to 3 blocks preferred (blocks a-c)]</u>

NOTE: This section is for the LYMPH NODE BLOCK matrix. Only positive lymph nodes found at the time of the prostatectomy should be entered into the matrix. <u>Lymph nodes removed at later dates should be entered in the METASTASIS BLOCK MATRIX</u>.

85a.Lymph Node Block Matrix #1: Neoplastic Block number:
Accession and Block # (i.e. SP-91-645-1A):

__ __ __ - __ __ - __ __ __ __ __ - __ __ __ __

86a.Lymph Node Block Matrix #1: Size of Largest Individual Nodule of Invasive Cancer (in cm)

__ __ __ . __ cm (0.x – 999, -1 = unknown)

87a.Lymph Node Block Matrix #1: Presence of Extracapsular Extension (ECE): (Circle One)

Yes No Unknown

==

85b.Lymph Node Block Matrix #2: Neoplastic Block number:
Accession and Block # (i.e. SP-91-645-1A):

__ __ __ - __ __ - __ __ __ __ __ - __ __ __ __

86b.Lymph Node Block Matrix #2: Size of Largest Individual Nodule of Invasive Cancer (in cm)

__ __ __ . __ cm (0.x – 999, -1 = unknown)

87b.Lymph Node Block Matrix #2: Presence of Extracapsular Extension (ECE): (Circle One)

Yes No Unknown

==

85c.Lymph Node Block Matrix #3: Neoplastic Block number:
Accession and Block # (i.e. SP-91-645-1A):

__ __ __ - __ __ - __ __ __ __ __ - __ __ __ __

86c.Lymph Node Block Matrix #3: Size of Largest Individual Nodule of Invasive Cancer (in cm)

__ __ __ . __ cm (0.x – 999, -1 = unknown)

87c.Lymph Node Block Matrix #3: Presence of Extracapsular Extension (ECE): (Circle One)

Yes No Unknown

RECURRENCE / METASTATSIS STATUS:

The Recurrence / Metastasis Status data elements are separated into three categories:

1) **Biochemical recurrence calls using the PSA recurrence algorithm developed by the CPCTR.**
2) **Clinically verified Tissue recurrence / metastasis for cases, which do not have blocks available for the Resource.**
3) **Metastatic Tissue block matrix for cases that have tissue available through the Resource from anatomical sites that show recurrence / metastasis of prostate cancer.**

BIOCHEMICAL RECURRENCE: (calculated values)

The biochemical recurrence calls are made using the PSA recurrence algorithm developed by the CPCTR. Further details on the algorithm used can be received by contacting the Resource.
NOTE: Data for #88-90 can be filled in manually from the IMS file for Institutional record. These fields DO NOT need to be captured locally.

88.Biochemical Recurrence Status:

Value	Value Description
**Never Disease Free**	Residual tumor present
**No recurrence**	No recurrence
**PSA recurrence**	Recurred based on CPCTR PSA recurrence algorithm
**Unknown**	Call cannot be determined using the algorithm

89.Recurrence PSA value:

__ __ __ __ . __ [>0.0; 9999.0, -1 (unknown)]

90.Date of Biochemical Recurrence Status:

__ __ / __ __ __ __ (MM / YYYY)

CLINICALLY VERIFIED TISSUE RECURRENCE/METASTASIS:

These cases are those that do not have tissue blocks available, but are known clinically to have a recurrence/metastasis.
NOTE: Verification for clinical recurrence can be via radiology imaging, biopsy/resection, or surgery. However, a clinician's note indicating recurrence in a specific distant site would be sufficient. However this does not include notes of recurrence because of PSA (Biochemical), note must say "bone, liver, etc. mets".

91.Tissue Recurrence/Metastasis: Distant site 1 (Circle One)

Bladder	Blood	Bone	Bone Marrow
Brain	Carcinomatosis	CNS	Generalized
Liver	Local extension	Lung	Peritoneum
Rectum	Lymph Nodes (Distant)		Lymph Nodes (Regional)
Skin	None	Other (NOS)	Unknown

92.Date of Tissue Recurrence/Metastasis: Distant site 1

__ __ / __ __ __ __ (MM/YYYY)

==

93.Tissue Recurrence/Metastasis: Distant site 2 (Circle One)

Bladder	Blood	Bone	Bone Marrow
Brain	Carcinomatosis	CNS	Generalized
Liver	Local extension	Lung	Peritoneum
Rectum	Lymph Nodes (Distant)		Lymph Nodes (Regional)
Skin	None	Other (NOS)	Unknown

94.Date of Tissue Recurrence/Metastasis: Distant site 2

__ __ / __ __ __ __ (MM/YYYY)

==

95.Tissue Recurrence / Metastasis: Distant site 3 (Circle One)

Bladder	Blood	Bone	Bone Marrow
Brain	Carcinomatosis	CNS	Generalized
Liver	Local extension	Lung	Peritoneum
Rectum	Lymph Nodes (Distant)		Lymph Nodes (Regional)
Skin	None	Other (NOS)	Unknown

96.Date of Tissue Recurrence / Metastasis: Distant site 3

__ __ / __ __ __ __ (MM / YYYY)

==

97.General Comments for Clinically Verified Tissue Recurrence / Metastasis:

____
____
____
____

METASTATIC TISSUE BLOCK MATRIX: (Items #98-108 are multiple entry fields):

-Enter as many blocks available (Three blocks are preferred).
-If multiple metastatic sites are present, then enter at least 1 block from each site.
-Try to include at least one block of normal tissue from the same site if possible. If it is not possible to find a completely normal block then include one with minimal amounts of carcinoma.
-The first 3 metastatic blocks matrix are included as *Blocks a, b, c.* (Print additional forms if necessary)

98a.Metastatic Block Matrix #1: Block number

Accession and Block # (i.e. SP-91-645-1A):

__ __ __ - __ __ - __ __ __ __ __ - __ __ __ __

99a.Metastatic Block Matrix #1: Specimen source (Circle One)

Biopsy **Resection** **Fine Needle Aspirate**

Other **Unknown**

100a.Metastatic Block Matrix #1: Tissue Type (Circle One)

Bladder **Blood** **Bone** **Bone Marrow**

Brain **Carcinomatosis** **CNS** **Generalized**

Liver **Local extension** **Lung** **Peritoneum**

Rectum **Lymph Nodes (Distant)** **Lymph Nodes (Regional)**

Skin **None** **Other (NOS)** **Unknown**

101a.Metastatic Block Matrix #1: Most Prominent Histological Type of Invasive Cancer (Circle One)

Adenocarcinoma NOS (aka acinar) Undifferentiated non-small cell carcinoma

Mucinous adenocarcinoma Signet ring adenocarcinoma Lymphoma

Transitional (not primary) Ductal adenocarcinoma Neuroendocrine carcinoma

Small cell anaplastic carcinoma Sarcomatoid carcinoma Basal cell carcinoma

Squamous or adenosquamous carcinoma Mesenchymal tumor (NOS)

PIN only Unknown Other (specify in comments)

Comments:____
____

102a.Metastatic Block Matrix #1: Size of Largest Individual Nodule of Invasive Cancer (in cm)

__ __ __ . __ cm (0.x – 999, -1 = unknown)

103a.Metastatic Block Matrix #1: Percentage of Tissue Occupied by Tumor (Circle One)

<5%   5 to 25%   >25%   Unknown

104a.Metastatic Block Matrix #1: Presence of Therapy effects (Circle One)

Yes   No   Unknown

105a.Metastatic Block Matrix #1: Non-Neoplastic Block number:
Accession and Block # (i.e. SP-91-645-1A):

__ __ __ - __ __ - __ __ __ __ __ - __ __ __ __

106a.Metastatic Block Matrix #1: Type of Block(s) available (Circle One)

Paraffin only   Frozen only   Paraffin and frozen

107a.Date of Metastatic Block Matrix #1:

__ __ / __ __ __ __ (MM/YYYY)

108a.General Comments Section for Metastatic Block Matrix #1:

____
____
____
____
____

==

98b.Metastatic Block Matrix #2: Block number

Accession and Block # (i.e. SP-91-645-1A):

__ __ __ - __ __ - __ __ __ __ __ - __ __ __ __

99b.Metastatic Block Matrix #2: Specimen source (Circle One)

Biopsy **Resection** **Fine Needle Aspirate**

Other **Unknown**

100b.Metastatic Block Matrix #2: Tissue Type (Circle One)

Bladder **Blood** **Bone** **Bone Marrow**

Brain **Carcinomatosis** **CNS** **Generalized**

Liver **Local extension** **Lung** **Peritoneum**

Rectum **Lymph Nodes (Distant)** **Lymph Nodes (Regional)**

Skin **None** **Other (NOS)** **Unknown**

101b.Metastatic Block Matrix #2: Most Prominent Histological Type of Invasive Cancer (Circle One)

Adenocarcinoma NOS (aka acinar) Undifferentiated non-small cell carcinoma

Mucinous adenocarcinoma Signet ring adenocarcinoma Lymphoma

Transitional (not primary) Ductal adenocarcinoma Neuroendocrine carcinoma

Small cell anaplastic carcinoma Sarcomatoid carcinoma Basal cell carcinoma

Squamous or adenosquamous carcinoma Mesenchymal tumor (NOS)

PIN only Unknown Other (specify in comments)

Comments:____
____

102b.Metastatic Block Matrix #2: Size of Largest Individual Nodule of Invasive Cancer (in cm)

__ __ __ . __ **cm (0.x – 999, -1 = unknown)**

103b.Metastatic Block Matrix #2: Percentage of Tissue Occupied by Tumor (Circle One)

<5%　　5 to 25%　　>25%　　Unknown

104b.Metastatic Block Matrix #2: Presence of Therapy effects (Circle One)

Yes　　No　　Unknown

105b.Metastatic Block Matrix #2: Non-Neoplastic Block number:
Accession and Block # (i.e. SP-91-645-1A):

__ __ __ - __ __ - __ __ __ __ __ - __ __ __ __

106b.Metastatic Block Matrix #2: Type of Block(s) available (Circle One)

Paraffin only　Frozen only　　Paraffin and frozen

107b.Date of Metastatic Block Matrix #2:

__ __ / __ __ __ __ **(MM/YYYY)**

108b.General Comments Section for Metastatic Block Matrix #2:

____
____
____
____
____

==

98c.Metastatic Block Matrix #3: Block number

Accession and Block # (i.e. SP-91-645-1A):

__ __ __ - __ __ - __ __ __ __ __ - __ __ __ __

99c.Metastatic Block Matrix #3: Specimen source (Circle One)

Biopsy **Resection** **Fine Needle Aspirate**

Other **Unknown**

100c.Metastatic Block Matrix #3: Tissue Type (Circle One)

Bladder **Blood** **Bone** **Bone Marrow**

Brain **Carcinomatosis** **CNS** **Generalized**

Liver **Local extension** **Lung** **Peritoneum**

Rectum **Lymph Nodes (Distant)** **Lymph Nodes (Regional)**

Skin **None** **Other (NOS)** **Unknown**

101c.Metastatic Block Matrix #3: Most Prominent Histological Type of Invasive Cancer (Circle One)

Adenocarcinoma NOS (aka acinar) Undifferentiated non-small cell carcinoma

Mucinous adenocarcinoma Signet ring adenocarcinoma Lymphoma

Transitional (not primary) Ductal adenocarcinoma Neuroendocrine carcinoma

Small cell anaplastic carcinoma Sarcomatoid carcinoma Basal cell carcinoma

Squamous or adenosquamous carcinoma Mesenchymal tumor (NOS)

PIN only Unknown Other (specify in comments)

Comments:____
____

102c.Metastatic Block Matrix #3: Size of Largest Individual Nodule of Invasive Cancer (in cm)

__ __ __ . __ cm (0.x – 999, -1 = unknown)

103c.Metastatic Block Matrix #3: Percentage of Tissue Occupied by Tumor (Circle One)

<5%   5 to 25%   >25%   Unknown

104c.Metastatic Block Matrix #3: Presence of Therapy effects (Circle One)

Yes   No   Unknown

105c.Metastatic Block Matrix #3: Non-Neoplastic Block number:
Accession and Block # (i.e. SP-91-645-1A):

__ __ __ - __ __ - __ __ __ __ __ - __ __ __ __

106c.Metastatic Block Matrix #3: Type of Block(s) available (Circle One)

Paraffin only Frozen only   Paraffin and frozen

107c.Date of Metastatic Block Matrix #3:

__ __ / __ __ __ __ **(MM/YYYY)**

108c.General Comments Section for Metastatic Block Matrix #3:
____
____
____
____
____

STAGING

Pathological and Clinical Staging will be based on using the AJCC Manual for Staging of Cancer, 5th Ed.

PATHOLOGICAL STAGING:

109. pT Stage** (Circle One)

pT0 **pT1** **pT1a** **pT1b** **pT1c**

pT2 **pT2a** **pT2b** **pT3** **pT3a** **pT3b**

pT4 **pTX**

Value	Value Description
pT0	No evidence of primary tumor
pT1	Clinically inapparent tumor not palpable nor visible by imaging
pT1a	Tumor incidental histologic finding in 5% or less of tissue resected
pT1b	Tumor incidental histologic finding in more than 5% of tissue resected
pT1c	Tumor identified by needle biopsy (e.g., because of elevated PSA)
pT2	Tumor confined within prostate*
pT2a	Tumor involves 1 lobe
pT2b	Tumor involves both lobes
pT3	Tumor extends through the prostatic capsule**
pT3a	Extracapsular extension (unilateral or bilateral)
pT3b	Tumor invades seminal vesicle(s)
pT4	Tumor is fixed or invades adjacent structures other than seminal vesicles
pTX	Primary tumor cannot be assessed

110. pN Stage** (Circle One)

pN0 **pN1** **pNX**

Value	Value Description
pN0	No regional lymph node metastasis
pN1	Metastasis in regional lymph node or nodes
pNX	Regional lymph nodes cannot be assessed

111. pM Stage** (Circle One)

pM0 **pM1** **pM1a** **pM1b** **pM1c** **pMX**

Value	Value Description
pM0	No distant metastasis
pM1	Distant metastasis
pM1a	Non-regional lymph node(s)
pM1b	Bone(s)
pM1c	Other site(s)*
pMX	Distant metastasis cannot be assessed

CLINICAL STAGING:

112. cT Stage (Circle One)

T0 **T1** **T1a** **T1b** **T1c**

T2 **T2a** **T2b** **T3** **T3a** **T3b**

T4 **TX**

Value	Value Description
T0	No evidence of primary tumor
T1	Clinically inapparent tumor not palpable nor visible by imaging
T1a	Tumor incidental histologic finding in 5% or less of tissue resected
T1b	Tumor incidental histologic finding in more than 5% of tissue resected
T1c	Tumor identified by needle biopsy (e.g., because of elevated PSA)
T2	Tumor confined within prostate*
T2a	Tumor involves 1 lobe
T2b	Tumor involves both lobes
T3	Tumor extends through the prostatic capsule**
T3a	Extracapsular extension (unilateral or bilateral)
T3b	Tumor invades seminal vesicle(s)
T4	Tumor is fixed or invades adjacent structures other than seminal vesicles
TX	Primary tumor cannot be assessed

113. cN Stage (Circle One)

N0 **N1** **NX**

Value	Value Description
N0	No regional lymph node metastasis
N1	Metastasis in regional lymph node or nodes
NX	Regional lymph nodes cannot be assessed

114. cM Stage (Circle One)

M0 **M1** **M1a** **M1b** **M1c** **MX**

Value	Value Description
M0	No distant metastasis
M1	Distant metastasis
M1a	Non-regional lymph node(s)
M1b	Bone(s)
M1c	Other site(s)*
MX	Distant metastasis cannot be assessed

115. General Staging Comments: ______________________________
____

VITAL STATUS/FOLLOW UP DATE

Record the patient's vital status and most recent follow up date.
NOTE: If an attempt was made to follow up the patient history but no new data was collected since the last follow up, make a note in the *final comments* that an attempt was made on a specific date, and that the case may be "lost to follow up".

Vital Status/Follow Up:

116. Date Last Known Alive*:

__ __ / __ __ __ __ (MM/YYYY)

117. Date of Death:

__ __ / __ __ __ __ (MM/YYYY)

118. Vital Status*: (Circle One)

Alive **Dead** **Lost to follow up**

Dead with Warm Autopsy

119. Final Comments:

____
____
____
____
____

THERAPY MATRIX (Items #120-123 are multiple entry fields): Record the patient's treatment history.

- -Enter as many treatment information available.
- -If therapy event was part of initial planned treatment, please mark appropriate data field.
- -The data fields for the first 3 therapy events are included as *Therapy matrix a, b, c.* (Print additional forms if necessary)
- -"*Per Initial Treatment Plan*" is defined as: If there is no treatment plan, established protocol, or management guidelines, and consultation with a physician advisor is not possible, use the principle: 'initial treatment must begin within four months of the date of initial diagnosis.

120a. Therapy matrix: Type of Therapy: (Circle One)

Brachytherapy Chemotherapy Cryotherapy Watchful waiting

External Beam Radiation Radiation Therapy, NOS Immunotherapy

Hormone Therapy, NOS Medical hormone suppression

Alternative Therapy (specify in comments) Surgical orchiectomy

Experimental (specify in comments)

121a. Therapy matrix: Therapy Start Date

__ __ / __ __ __ __ (MM/YYYY)

122a. Therapy matrix: Per Initial Treatment Plan? ** (Circle One)

Yes No Unknown

123a. Therapy Matrix Comments:

____
____
____
==

120b. Therapy matrix: Type of Therapy: (Circle One)

Brachytherapy Chemotherapy Cryotherapy Watchful waiting

External Beam Radiation Radiation Therapy, NOS Immunotherapy

Hormone Therapy, NOS Medical hormone suppression

Alternative Therapy (specify in comments) Surgical orchiectomy

Experimental (specify in comments)

121b. Therapy matrix: Therapy Start Date

__ __ / __ __ __ __ (MM/YYYY)

122b. Therapy matrix: Per Initial Treatment Plan? ** (Circle One)

(If there is no treatment plan, established protocol, or management guidelines, and consultation with a physician advisor is not possible, use the principle: 'initial treatment must begin within four months of the date of initial diagnosis.)

Yes **No** **Unknown**

123b. Therapy Matrix Comments:

____
____
____

==

120c. Therapy matrix: Type of Therapy: (Circle One)

Brachytherapy **Chemotherapy** **Cryotherapy** **Watchful waiting**

External Beam Radiation **Radiation Therapy, NOS** **Immunotherapy**

Hormone Therapy, NOS **Medical hormone suppression**

Alternative Therapy (specify in comments) **Surgical orchiectomy**

Experimental (specify in comments)

121c. Therapy matrix: Therapy Start Date

__ __ / __ __ __ __ (MM/YYYY)

122c. Therapy matrix: Per Initial Treatment Plan? ** (Circle One)

Yes **No** **Unknown**

123c. Therapy Matrix Comments:

____
____

Additional overall Therapy comments:

124. Comments on Therapy Response:

____
____

125. General Overall Comments on Therapy:

____
____

NEEDLE BIOPSY BLOCK MATRIX: [Items #126-134 are multiple entry fields. <u>Up to 5 blocks preferred (blocks a-e)]</u>

- **Here are the recommended criteria for needle biopsy:**

1) Can include one block or more (up to 5)
2) Must at least include one neoplastic block and classify according to the needle biopsy matrix.
NOTE: for biopsy-only cases, it is a requirement to enter the "subsequent prostatectomy" field in the biopsy attribute section. "<u>Biopsy-Only" cases</u> are cases due to advance disease or other reasons that did NOT lead to a prostatectomy.

126a. Biopsy Matrix: Block number

Accession and Block # (i.e. SP-91-645-1A):

__ __ __ - __ __ - __ __ __ __ __ - __ __ __ __

127a. Biopsy Matrix: Most Prominent Histological Type of Invasive Cancer: (Circle One)

Adenocarcinoma NOS (aka acinar) Undifferentiated non-small cell carcinoma

Mucinous adenocarcinoma Signet ring adenocarcinoma Lymphoma

Transitional (not primary) Ductal adenocarcinoma Neuroendocrine carcinoma

Small cell anaplastic carcinoma Sarcomatoid carcinoma Basal cell carcinoma

Squamous or adenosquamous carcinoma Mesenchymal tumor (NOS)

PIN only Unknown Other (specify in comments)

128a. Biopsy Matrix: Primary Gleason Grade: (Circle One)

Gleason grade: 1 2 3 4 5

not adenocarcinoma not primary tumor Unknown PIN only

129a. Biopsy Matrix: Secondary Gleason Grade: (Circle One)

Gleason grade: 1 2 3 4 5

not adenocarcinoma not primary tumor Unknown PIN only

130a. Biopsy Matrix: Size of Largest Individual Nodule of Invasive Cancer (in cm)

__ __ . __ cm (-1=unknown, 0.0 – 99.9 cm)

(131a-133a) BIOPSY BLOCK ATTRIBUTES: Presence of: High Grade Prostatic Intraepithelial Neoplasia (PIN); Perineural Invasion (PN); Angiolymphatic Invasion (AL). (circle all that apply)

PIN PN AL None

134a. Biopsy Matrix: block comments

____
____
____
____

==

126b. Biopsy Matrix: Block number

Accession and Block # (i.e. SP-91-645-1A):

__ __ __ - __ __ - __ __ __ __ __ - __ __ __ __

127b. Biopsy Matrix: Most Prominent Histological Type of Invasive Cancer: (Circle One)

Adenocarcinoma NOS (aka acinar) Undifferentiated non-small cell carcinoma

Mucinous adenocarcinoma Signet ring adenocarcinoma Lymphoma

Transitional (not primary) Ductal adenocarcinoma Neuroendocrine carcinoma

Small cell anaplastic carcinoma Sarcomatoid carcinoma Basal cell carcinoma

Squamous or adenosquamous carcinoma Mesenchymal tumor (NOS)

PIN only Unknown Other (specify in comments)

128b. Biopsy Matrix: Primary Gleason Grade: (Circle One)

Gleason grade: 1 2 3 4 5

not adenocarcinoma not primary tumor Unknown PIN only

129b. Biopsy Matrix: Secondary Gleason Grade: (Circle One)

Gleason grade: 1 2 3 4 5

not adenocarcinoma not primary tumor Unknown PIN only

130b. Biopsy Matrix: Size of Largest Individual Nodule of Invasive Cancer (in cm)

__ __ . __ cm (-1=unknown, 0.0 – 99.9 cm)

(131b-133b) BIOPSY BLOCK ATTRIBUTES: Presence of:
High Grade Prostatic Intraepithelial Neoplasia (PIN); Perineural Invasion (PN); Angiolymphatic Invasion (AL). (circle all that apply)

PIN PN AL None

134b. Biopsy Matrix: block comments

____
____
____
____

==

126c. Biopsy Matrix: Block number

Accession and Block # (i.e. SP-91-645-1A):

__ __ __ - __ __ - __ __ __ __ __ - __ __ __ __

127c. Biopsy Matrix: Most Prominent Histological Type of Invasive Cancer: (Circle One)

Adenocarcinoma NOS (aka acinar) Undifferentiated non-small cell carcinoma

Mucinous adenocarcinoma Signet ring adenocarcinoma Lymphoma

Transitional (not primary) Ductal adenocarcinoma Neuroendocrine carcinoma

Small cell anaplastic carcinoma Sarcomatoid carcinoma Basal cell carcinoma

Squamous or adenosquamous carcinoma Mesenchymal tumor (NOS)

PIN only Unknown Other (specify in comments)

128c. Biopsy Matrix: Primary Gleason Grade: (Circle One)

Gleason grade: 1 2 3 4 5

not adenocarcinoma not primary tumor Unknown PIN only

129c. Biopsy Matrix: Secondary Gleason Grade: (Circle One)

Gleason grade: 1 2 3 4 5

not adenocarcinoma not primary tumor Unknown PIN only

130c. Biopsy Matrix: Size of Largest Individual Nodule of Invasive Cancer (in cm)

__ __ . __ cm (-1=unknown, 0.0 – 99.9 cm)

(131c-133c) BIOPSY BLOCK ATTRIBUTES: Presence of: High Grade Prostatic Intraepithelial Neoplasia (PIN); Perineural Invasion (PN); Angiolymphatic Invasion (AL). (circle all that apply)

PIN PN AL None

134c. Biopsy Matrix: block comments

____
____
____
____

==

126d. Biopsy Matrix: Block number

Accession and Block # (i.e. SP-91-645-1A):

__ __ __ - __ __ - __ __ __ __ __ - __ __ __ __

127d. Biopsy Matrix: Most Prominent Histological Type of Invasive Cancer: (Circle One)

Adenocarcinoma NOS (aka acinar) Undifferentiated non-small cell carcinoma

Mucinous adenocarcinoma Signet ring adenocarcinoma Lymphoma

Transitional (not primary) Ductal adenocarcinoma Neuroendocrine carcinoma

Small cell anaplastic carcinoma Sarcomatoid carcinoma Basal cell carcinoma

Squamous or adenosquamous carcinoma Mesenchymal tumor (NOS)

PIN only Unknown Other (specify in comments)

128d. Biopsy Matrix: Primary Gleason Grade: (Circle One)

Gleason grade: 1 2 3 4 5

not adenocarcinoma not primary tumor Unknown PIN only

129d. Biopsy Matrix: Secondary Gleason Grade: (Circle One)

Gleason grade: 1 2 3 4 5

not adenocarcinoma not primary tumor Unknown PIN only

130d. Biopsy Matrix: Size of Largest Individual Nodule of Invasive Cancer (in cm)

__ __ . __ cm (-1=unknown, 0.0 – 99.9 cm)

(131d-133d) BIOPSY BLOCK ATTRIBUTES: Presence of: High Grade Prostatic Intraepithelial Neoplasia (PIN); Perineural Invasion (PN); Angiolymphatic Invasion (AL). (circle all that apply)

PIN PN AL None

134d. Biopsy Matrix: block comments

____
____
____
____

==

126e. Biopsy Matrix: Block number

Accession and Block # (i.e. SP-91-645-1A):

__ __ __ - __ __ - __ __ __ __ __ - __ __ __ __

127e. Biopsy Matrix: Most Prominent Histological Type of Invasive Cancer: (Circle One)

Adenocarcinoma NOS (aka acinar)   Undifferentiated non-small cell carcinoma

Mucinous adenocarcinoma   Signet ring adenocarcinoma   Lymphoma

Transitional (not primary)   Ductal adenocarcinoma   Neuroendocrine carcinoma

Small cell anaplastic carcinoma   Sarcomatoid carcinoma   Basal cell carcinoma

Squamous or adenosquamous carcinoma   Mesenchymal tumor (NOS)

PIN only   Unknown   Other (specify in comments)

128e. Biopsy Matrix: Primary Gleason Grade: (Circle One)

Gleason grade: 1   2   3   4   5

not adenocarcinoma   not primary tumor   Unknown   PIN only

129e. Biopsy Matrix: Secondary Gleason Grade: (Circle One)

Gleason grade: 1   2   3   4   5

not adenocarcinoma   not primary tumor   Unknown   PIN only

130e. Biopsy Matrix: Size of Largest Individual Nodule of Invasive Cancer (in cm)

__ __ . __ cm (-1=unknown, 0.0 – 99.9 cm)

(131e-133e) BIOPSY BLOCK ATTRIBUTES: Presence of: High Grade Prostatic Intraepithelial Neoplasia (PIN); Perineural Invasion (PN); Angiolymphatic Invasion (AL). (circle all that apply)

PIN   PN   AL   None

134e. Biopsy Matrix: block comments

____
____
____
____

OVERALL NEEDLE BIOPSY ATTRIBUTES:

- **Enter the overall characteristics of the biopsy.**
- **For "biopsy-only" cases, the subsequent prostatectomy field is a required field.**
  NOTE: "Biopsy-Only" cases are cases due to advance disease or other reasons that did NOT lead to a prostatectomy.

135. Biopsy: Most Prominent Histological Type of Invasive Cancer (Circle One)

Adenocarcinoma NOS (aka acinar)   Undifferentiated non-small cell carcinoma

Mucinous adenocarcinoma   Signet ring adenocarcinoma   Lymphoma

Transitional (not primary)   Ductal adenocarcinoma   Neuroendocrine carcinoma

Small cell anaplastic carcinoma   Sarcomatoid carcinoma   Basal cell carcinoma

Squamous or adenosquamous carcinoma   Mesenchymal tumor (NOS)

PIN only   Unknown   Other (specify in comments)

136. Biopsy: Primary Gleason Grade: (Circle One)

Gleason grade: 1   2   3   4   5

not adenocarcinoma   not primary tumor   Unknown   PIN only

137. Biopsy: Secondary Gleason Grade: (Circle One)

Gleason grade: 1   2   3   4   5

not adenocarcinoma   not primary tumor   Unknown   PIN only

138. Biopsy: Gleason Sum Score: (Circle One)

Gleason score 2   Gleason score 3   Gleason score 4

Gleason score 5   Gleason score 6   Gleason score 7

Gleason score 8   Gleason score 9   Gleason score 10

not adenocarcinoma   not primary tumor   unknown

139. Biopsy: Percentage of Gleason 4/5 grade (in all of 6's and 7's)

__ __ __ % (0.x – 100 %, -1 = unknown)

140. Percentage of Biopsy Occupied by Tumor: (Circle One)

<5%   5 to 25%   >25%   Unknown

141. Biopsy: Presence of High Grade Prostatic Intraepithelial Neoplasia: (Circle One)

Yes - # of foci unknown   **Yes – 1 or 2 foci in region of tumor**

Yes – 1 or 2 foci away from tumor   **Yes – multifocal**

No   **Unknown**

142. Biopsy: Non-Neoplastic Block #1:
Accession and Block # (i.e. SP-91-645-1A):

__ __ __ - __ __ - __ __ __ __ __ - __ __ __ __

143. Subsequent Prostatectomy: (Circle One)**
Definition: **Specifies whether a subsequent prostatectomy occurred. This field will help clarify cases where only biopsy specimens are available.**

Yes   No   Unknown

144. Subsequent Prostatectomy comments:

____
____
____
____

145. General Comments on Biopsy:

____
____
____
____
